# Microbiome Dynamics: A Paradigm Shift in Combatting Infectious Diseases

**DOI:** 10.3390/jpm14020217

**Published:** 2024-02-18

**Authors:** Mohamed Kamel, Sami Aleya, Majed Alsubih, Lotfi Aleya

**Affiliations:** 1 Department of Medicine and Infectious Diseases, Faculty of Veterinary Medicine, Cairo University, Giza 11221, Egypt; 2 Faculty of Medecine, Université de Bourgogne Franche-Comté, Hauts-du-Chazal, 25030 Besançon, France; sami.aleya@edu.univ-fcomte.fr; 3 Department of Civil Engineering, King Khalid University, Guraiger, Abha 62529, Saudi Arabia; malsubih@kku.edu.sa; 4 Laboratoire de Chrono-Environnement, Université de Bourgogne Franche-Comté, UMR CNRS 6249, La Bouloie, 25030 Besançon, France; lotfi.aleya@univ-fcomte.fr

**Keywords:** infectious diseases, human microbiome, microbiome-targeted therapies, treatment approaches, host–pathogen interactions

## Abstract

Infectious diseases have long posed a significant threat to global health and require constant innovation in treatment approaches. However, recent groundbreaking research has shed light on a previously overlooked player in the pathogenesis of disease—the human microbiome. This review article addresses the intricate relationship between the microbiome and infectious diseases and unravels its role as a crucial mediator of host–pathogen interactions. We explore the remarkable potential of harnessing this dynamic ecosystem to develop innovative treatment strategies that could revolutionize the management of infectious diseases. By exploring the latest advances and emerging trends, this review aims to provide a new perspective on combating infectious diseases by targeting the microbiome.

## 1. Introduction

The human body is inhabited by trillions of microorganisms that collectively form what is known as the microbiome [1,2,3]. This intricate ecosystem consists of bacteria, viruses, fungi, and other microbes that reside on various surfaces, such as the skin, mouth, gastrointestinal tract, and reproductive organs [1,2,3] (see Table 1). Over the past decade, there has been a growing interest in understanding the role of the microbiome in human health and disease [1,2,3]. One area that has garnered significant attention is the impact of the microbiome on infectious diseases.

Infectious diseases remain a major global health challenge, causing significant morbidity and mortality worldwide. Traditionally, these diseases have been attributed solely to pathogens, such as bacteria, viruses, and parasites. However, emerging research has revealed that the microbiome plays a crucial role in shaping host susceptibility to infections and influencing disease outcomes [4,5]. The microbiome interacts with pathogens directly or indirectly through various mechanisms, including competition for resources, production of antimicrobial substances, modulation of immune responses, and alteration in the local microenvironment [6].

Understanding how the microbiome influences infectious diseases is not only important for unraveling the complex interplay between microbes and their hosts but also holds great promise for the development of novel therapeutic strategies. By elucidating the underlying mechanisms by which the microbiome affects pathogenesis, we can identify potential targets for intervention and formulate innovative treatment approaches [7]. Furthermore, the composition of the microbiome can serve as a diagnostic tool for predicting disease susceptibility, monitoring disease progression, and assessing treatment efficacy.

**Table 1:** Microbiota composition and potential impacts on health in different body sites.

Body Site	Dominant Phyla	Key Bacterial Genera	Functions	Potential Impacts on Health	References
Gut	Bacteroidetes, Firmicutes	*Bacteroides*, *Bifidobacterium*, *Clostridium*, *Faecalibacterium*, *Lactobacillus*	Digestion of dietary fibers, nutrient absorption, vitamin synthesis, immune system modulation	Obesity, diabetes, inflammatory bowel disease, cancer	[8,9]
Oral Cavity	Firmicutes, Bacteroidetes	*Streptococcus*, *Actinomyces*, *Prevotella*, *Porphyromonas*	Breakdown of dietary carbohydrates, fermentation, barrier against pathogens	Dental caries, periodontal disease, halitosis	[10]
Skin	Actinobacteria, Firmicutes, Proteobacteria	*Staphylococcus*, *Propionibacterium*, *Corynebacterium*, *Cutibacterium*	Sebum production, sweat decomposition, barrier against pathogens	Acne, eczema, psoriasis, wound healing	[11]
Vagina	Firmicutes, Bacteroidetes	*Lactobacillus*, *Bifidobacterium*, *Gardnerella*, *Prevotella*	Maintain optimal pH balance, prevent colonization by pathogens	Bacterial vaginosis, yeast infections, preterm birth	[12,13]
Respiratory Tract	Firmicutes, Bacteroidetes	*Streptococcus*, *Haemophilus*, *Staphylococcus*, *Prevotella*	Gas exchange, immune system modulation, barrier against pathogens	Pneumonia, asthma, cystic fibro	[14,15]

The objectives of this review are to comprehensively explore the current understanding of the role of the microbiome in infectious disease pathogenesis and to highlight potential treatment strategies that target the microbiome. Specifically, we will examine recent findings on how alterations in the composition and function of the microbiome contribute to the development and progression of infectious diseases. Additionally, we will discuss various approaches aimed at modulating the microbiome to prevent or treat infections, including probiotics, prebiotics, fecal microbiota transplantation, and targeted antimicrobial therapies.

By synthesizing existing knowledge and discussing recent advancements in the field, this review aims to provide a comprehensive overview of the importance of the microbiome in infectious diseases. By shedding light on the intricate relationship between microbes and their hosts, we stimulate further research in this area and contribute to the development of effective strategies for preventing and treating infectious diseases. Understanding the role of the microbiome in infectious diseases has the potential to revolutionize our approach to healthcare and significantly improve patient outcomes in the future.

## 2. Microbiome Dysbiosis and Infectious Diseases

The human microbiome, comprising trillions of microbes residing in and on our bodies, plays a crucial role in maintaining our health. The complex microbiota community interacts with our immune system and influences various physiological processes, including digestion, metabolism, and protection against pathogens [2,3,16,17]. When this delicate balance is disrupted, a condition known as dysbiosis occurs. Dysbiosis is characterized by imbalances in the microbiome and has been implicated in the development and progression of various infectious diseases. Dysbiosis has been associated with conditions such as inflammatory bowel disease, irritable bowel syndrome, obesity, metabolic syndrome, allergies, asthma, autoimmune diseases, and mental health disorders [18,19].

Infectious diseases are caused by pathogens such as bacteria, viruses, and parasites. While it is well established that these pathogens directly contribute to the development of infectious diseases, emerging evidence suggests that dysbiosis of the microbiome can also play a significant role. Dysbiosis can alter the composition and function of the microbiota, leading to a compromised immune response and increased susceptibility to infection [20]. Several infectious diseases have been associated with microbiome dysbiosis. One such example is *Clostridioides* (*C*) *difficile* infection (CDI), a potentially life-threatening condition characterized by severe diarrhea. CDI commonly occurs following antibiotic treatment, which disrupts the normal gut microbiota and allows *C. difficile* to overgrow [21,22]. Studies have shown that individuals with a healthy and diverse microbiome are less susceptible to CDI compared with those with dysbiosis.

In addition to CDI, respiratory tract infections have also been linked to microbiome dysbiosis. Research indicates that alterations in the lung microbiota can predispose individuals to respiratory infections such as pneumonia and chronic obstructive pulmonary disease (COPD). Dysbiosis in the lung microbiota can weaken the respiratory defense mechanisms, making individuals more susceptible to pathogen colonization and infection [23,24].

Furthermore, sexually transmitted infections (STIs) have also been associated with microbiome dysbiosis. For instance, bacterial vaginosis (BV), a common vaginal infection, is characterized by an overgrowth of harmful bacteria and a decrease in beneficial *lactobacilli* [25]. This dysbiosis disrupts the vaginal ecosystem and increases the risk of acquiring STIs such as *Chlamydia trachomatis* and *Neisseria gonorrhoeae* [26].

Gastrointestinal infections, such as *Helicobacter pylori* infection and foodborne illnesses, have also been linked to dysbiosis [27,28,29]. *H. pylori* is a bacterium that colonizes the stomach and is associated with various gastrointestinal disorders, including gastric ulcers and stomach cancer. Dysbiosis of the gastric microbiome can influence *H. pylori* colonization and disease progression [30,31].

Moreover, dysbiosis has been implicated in the development of inflammatory bowel diseases (IBDs) such as Crohn’s disease and ulcerative colitis. These chronic conditions are characterized by inflammation of the gastrointestinal tract and are believed to result from an abnormal immune response triggered by dysbiosis [18,32]. Imbalances in the gut microbiota composition have been observed in individuals with IBD, suggesting a potential role for dysbiosis in disease pathogenesis.

Emerging evidence also suggests a link between dysbiosis and urinary tract infections (UTIs). The urinary tract is normally sterile; however, certain bacteria can disrupt this balance and cause UTIs. Dysbiosis of the urogenital microbiota may contribute to UTI susceptibility by altering the protective mechanisms that prevent bacterial colonization.

Furthermore, skin infections have also been associated with microbiome dysbiosis. Conditions such as acne, atopic dermatitis, and wound infections have been linked to alterations in the skin microbiota composition [33,34,35]. Dysbiosis of the skin microbiome can disrupt the natural defense mechanisms of the skin, allowing pathogens to colonize and cause infections [36].

Additionally, dysbiosis has been implicated in the development of oral infections such as dental caries (tooth decay) and periodontal diseases. The oral cavity harbors a diverse microbial community that helps maintain oral health [37]. Imbalances in this community can lead to an overgrowth of harmful bacteria, contributing to the development of oral infections.

Furthermore, dysbiosis of the vaginal microbiome has been associated with an increased risk of preterm birth and other pregnancy complications. Imbalances in vaginal microbial composition can lead to inflammation and an overgrowth of pathogenic bacteria, disrupting the delicate environment required for a healthy pregnancy [38,39,40].

## 3. Mechanisms of Microbiome-Mediated Pathogenesis

The microbiome, which consists of various bacteria, fungi, and viruses residing within and on our bodies, plays a crucial role in the development of infectious diseases [41]. This influence is exerted through a complex interplay of mechanisms. Firstly, the microbiome competes directly with pathogens for nutrients and space, effectively creating a barrier to infection. For instance, lactobacilli in the female genital tract produce lactic acid, inhibiting the growth of pathogenic yeasts and bacteria [42]. Similarly, gut bacteria metabolize dietary fibers to produce short-chain fatty acids, which nourish colonocytes and create an unfavorable environment for many pathogens. However, disturbances in the microbial ecosystem can facilitate pathogen invasion [43,44].

Secondly, the microbiome modulates host immune responses, either protecting against or promoting infectious diseases. Commensal microbes prime the immune system, promoting immune cell maturation, antimicrobial peptide production, and inflammation regulation [45,46]. For example, segmented filamentous bacteria in the gut stimulate the production of Th17 cells, which protect against bacterial and fungal infections [47,48,49]. On the other hand, some commensal bacteria downregulate immune responses or induce tolerance to prevent overreaction to harmless antigens, creating opportunities for pathogens to establish infections [50,51].

Additionally, commensal microorganisms produce antimicrobial compounds that inhibit or kill potential pathogens. These compounds include bacteriocins and substances like ethanol and acetic acid [52,53,54]. The microbiome’s production of these compounds contributes to colonization resistance, preventing opportunistic pathogens from gaining a foothold. However, antibiotic treatment that reduces beneficial microbes can diminish this protective effect [55,56].

The microbiome also influences the integrity of mucosal barriers, preventing pathogen invasion. Interactions between commensal microbes and host epithelial cells affect cell turnover, repair, and the production of mucus and tight junction proteins that maintain barrier function [57,58]. Disruptions in these interactions can lead to increased intestinal permeability, allowing pathogens easier access to tissues and contributing to inflammation and infection.

Furthermore, the microbiome indirectly affects pathogenesis by influencing other members of the microbial community. Commensal bacteria can alter local pH levels, produce metabolic byproducts that influence neighboring microbes’ growth and virulence factor expression, interfere with quorum sensing, or degrade virulence factors produced by pathogens [59,60,61]. The balance and composition of the microbiome are critical in determining whether pathogenic microbes can thrive and cause disease.

In addition to these mechanisms, the microbiome’s impact extends to microbial metabolites and host signaling pathways. Microbial metabolites such as short-chain fatty acids generated by gut bacteria can modulate immune responses and enhance gut barrier integrity [2,3,62,63,64]. Dysregulation of these metabolic pathways due to changes in the microbiome composition may increase the risk of infection [64,65].

The presence of certain microbiota can also influence pathogen virulence by suppressing gene expression or interfering with their ability to invade host tissues. Some commensal bacteria can sequester limiting nutrients like iron, reducing its availability to pathogens and limiting their growth [66,67,68]. Moreover, the microbiome can induce the expression of host-derived antimicrobial proteins that target specific aspects of pathogen physiology.

The microbiome can impact antibiotic efficacy and resistance as well. The microbiome can impact antibiotic efficacy by metabolizing antibiotics, disrupting the microbial balance, and promoting biofilm formation [69]. The diverse microbial community in our bodies can harbor genes that confer resistance to antibiotics. These genes can be transferred among bacteria through horizontal gene transfer, potentially leading to the emergence of antibiotic-resistant infections [70,71]. Additionally, antibiotics can have broad impacts on the microbiome, targeting not only the causative agent but also commensal organisms. Antibiotics disrupt the healthy balance of gut flora, leading to a reduction in diversity and changes in both populations and functions [72,73]. This disruption can also contribute to the spread of antibiotic resistance. Furthermore, the effects of antibiotics on the gut microbiota can persist for extended periods, even after the cessation of antibiotic use [72,73]. This collateral damage can lead to dysbiosis and the overgrowth of resistant pathogens.

Furthermore, the microbiome is involved in drug metabolism and can affect the pharmacokinetics of medications used to treat infections [74,75]. Microbial enzymes within the gut can activate or inactivate drugs, altering their effectiveness, affecting their absorption and distribution, and potentially interacting with other medications and toxicity profiles [76,77,78]. Optimal dosing and drug delivery strategies need to consider these interactions.

The microbiome also influences an individual’s susceptibility to infections through chronic diseases like diabetes, obesity, and IBD [79]. The human microbiome, particularly in the gut, plays a crucial role in protecting against infections and chronic inflammatory diseases by interacting with and regulating the immune system response. However, an imbalance in the gut microbiome can lead to impaired immune function and the development of conditions such as diabetes, obesity, and inflammatory bowel disease (IBD). These conditions, in turn, can further impact the health of the microbiome [80,81]. Changes in microbiome composition and function associated with these conditions can impair immune responses and mucosal barrier functions, potentially increasing vulnerability to pathogens.

In addition, the microbiome impacts host epigenetic regulation, modifying gene expression patterns without altering DNA sequences. Microbial metabolites and components can influence enzymes involved in DNA methylation and histone modification, altering gene expression profiles in host cells [1,82,83,84,85,86]. These epigenetic changes affect immune responses, inflammation, and susceptibility to infectious diseases.

The microbiome has a significant impact on the transmission and dissemination of infectious agents and antibiotic resistance genes by influencing competition with pathogens, modulating immune responses, enhancing barrier function, metabolizing nutrients, and training the immune system [87,88,89,90]. Pathogens can exploit the microbial community as a reservoir and means of transmission. For example, some bacteria can form biofilms within the microbiota, providing protection and a means of spreading to other individuals. Furthermore, the transfer of antibiotic resistance genes between commensal bacteria and pathogens can occur, contributing to the spread of drug-resistant infections [91,92]. Understanding these transmission dynamics is crucial for implementing effective strategies to control and prevent the spread of infectious diseases.

The composition and diversity of the microbiome can influence the efficacy of vaccines [93,94]. The diversity of the human microbiome is influenced by a wide range of factors, including age, sex, diet, physical activity, stress, host genetics, diseases, breastfeeding status, lifestyle, delivery mode, geographical and racial variations, temperature, medications, diseases, and bacteriophage [2,95,96,97,98,99,100]. Microbial diversity within the microbiome varies across different body sites and depends on external and internal factors [101,102,103,104]. Commensal microorganisms can interact with the immune system and influence its response to vaccination [105,106]. They can modulate the maturation and activation of immune cells, promote antibody production, and influence the development of immunological memory. Alterations in the microbiome composition, such as those induced by antibiotic use or dysbiosis, may affect vaccine responses and compromise their effectiveness [107,108,109]. Understanding how the microbiome influences vaccine efficacy can inform strategies to optimize immunization protocols and improve protection against infectious diseases.

The microbiome plays a crucial role in host metabolism and nutrient availability, indirectly influencing infectious disease pathogenesis. The gut microbiota community is involved in the digestion and absorption of nutrients, including vitamins and minerals essential for immune function [110]. Disruptions in the microbiome can lead to malabsorption or altered nutrient availability, impacting host health and immune responses. Furthermore, changes in microbial metabolism can produce metabolites that affect systemic processes such as glucose metabolism, lipid metabolism, and inflammation. These alterations can influence an individual’s susceptibility to infections and their ability to mount effective immune responses.

Early-life exposures and interactions with the microbiome can have long-lasting effects on host health and disease susceptibility. The establishment of a healthy microbiome during infancy is critical for immune system development and maturation [111,112]. Perturbations in early microbial colonization, such as through cesarean section delivery or formula feeding, have been associated with an increased risk of developing infections later in life [113]. The microbiome can also influence the development of immune tolerance and allergic sensitization [114]. Understanding the role of early-life microbial exposures in shaping host susceptibility to infectious diseases can inform interventions aimed at optimizing microbiome development and reducing disease burden.

The microbiome plays a crucial role in regulating inflammatory responses in the host. Commensal microorganisms help maintain a state of immune homeostasis by promoting the production of anti-inflammatory cytokines and inhibiting the release of pro-inflammatory cytokines [115,116]. This balance is essential for preventing excessive inflammation, which can contribute to tissue damage and promote the growth and spread of pathogens. Dysbiosis disrupts this delicate equilibrium, leading to chronic inflammation that may contribute to the pathogenesis of infectious diseases.

Commensal microorganisms communicate with host cells through various signaling pathways, influencing cellular processes and immune cell development. Microbial metabolites and cell surface molecules can bind to specific receptors on host cells, triggering signaling cascades that affect cellular functions. These interactions can impact the differentiation and maturation of immune cells, including T cells, B cells, and dendritic cells, thus influencing the overall immune response to infection [117,118,119]. Disruptions in these interactions, such as alterations in the microbiome composition or the loss of specific commensal species, can lead to immune dysregulation and increased susceptibility to infectious diseases.

Emerging evidence suggests a bidirectional communication between the gut microbiome and the central nervous system, known as the gut–brain axis. The microbiome can influence brain function and behavior through various mechanisms, including the production of neurotransmitters and metabolites that can cross the blood–brain barrier (BBB). This intricate connection has implications for infectious diseases, as alterations in the gut microbiome composition may influence host responses to pathogens via neuroimmune interactions [120,121]. Moreover, stress-induced changes in the gut microbiota can impact immune responses and increase susceptibility to infections, highlighting the role of the gut–brain axis in infectious disease pathogenesis [122,123,124]. Additionally, the microbiome has been found to influence infectious diseases involving the BBB through mechanisms such as metabolite production and immune system modulation. Certain bacteria-produced metabolites, like short-chain fatty acids, can affect BBB integrity and permeability [125]. Dysregulation of the gut microbiota can also impact immune responses within the CNS, making the BBB more susceptible to pathogen invasion [126,127]. Targeting the microbiome through interventions like probiotics or fecal microbiota transplantation shows promise for restoring BBB integrity and enhancing immune defenses. Understanding specific microbial interactions with BBB receptors may lead to targeted therapies for infectious diseases involving the BBB.

The microbiome and host metabolism have a mutually beneficial relationship. Commensal microorganisms contribute to host metabolism by assisting in the digestion and fermentation of complex dietary components such as fiber, producing metabolites that can be utilized by both the host and other members of the microbiome [128,129]. In turn, these microbial metabolic activities influence host metabolic pathways, including energy metabolism and lipid homeostasis [110,129]. Dysbiosis or alterations in dietary patterns can disrupt these metabolic interactions, potentially impacting host immune function and susceptibility to infections.

Host genetic factors play a role in shaping the composition and function of the microbiome, which can subsequently influence infectious disease pathogenesis [130,131]. Genetic variations can affect host–microbe interactions by influencing immune responses, mucosal barrier integrity, and susceptibility to dysbiosis [132]. Host genetic factors also impact the ability of commensal microorganisms to colonize specific niches within the body or produce antimicrobial substances that can inhibit pathogens [133,134]. Understanding the interplay between host genetics and the microbiome is crucial for unraveling individual variations in infection susceptibility and developing personalized approaches to prevent and treat infectious diseases.

The microbiome plays a crucial role in the development and function of mucosal immunity, particularly at sites such as the respiratory and gastrointestinal tracts. Commensal microorganisms contribute to the maturation and activation of immune cells within these mucosal tissues, including specialized cells such as M cells and secretory IgA-producing plasma cells [135,136]. These immune cells help defend against pathogens and maintain immune homeostasis. Disruptions in the microbiome can impair the development and function of mucosal immunity, leading to increased susceptibility to infections at these sites.

The microbiome engages in nutrient competition with pathogens, limiting the availability of essential nutrients required for pathogen growth and survival. Commensal microorganisms can outcompete pathogens for nutrients such as iron, zinc, and vitamins, thereby reducing their ability to establish infections [59,137]. For example, lactobacilli in the vaginal microbiota produce bacteriocins that can chelate iron, limiting its availability to pathogens [138]. These nutrient competition mechanisms contribute to colonization resistance and help protect against pathogen invasion and overgrowth.

The microbiome can influence host tissue repair and regeneration processes, which are essential for resolving infections and restoring tissue integrity. Commensal microorganisms can interact with host cells to promote wound healing, tissue remodeling, and regeneration [139,140]. They can modulate signaling pathways involved in cell proliferation, migration, and extracellular matrix production. Disruptions in the microbiome can impair these tissue repair processes, potentially prolonging infection duration or increasing the risk of tissue damage.

While much focus is on bacterial pathogens, the microbiome also influences antiviral immune responses. Commensal microorganisms shape the development and functionality of antiviral immune cells, such as natural killer (NK) cells and dendritic cells, as well as influence the production of interferons and other antiviral molecules [141,142,143]. The presence of a healthy and diverse microbiome can enhance antiviral defenses by promoting immune cell activation and modulating cytokine responses. Moreover, certain commensal bacteria have been found to directly inhibit viral replication through the production of antiviral substances or competition for viral attachment sites [144,145,146,147].

### Microbiome-Induced Changes in Host Barrier Permeability

The composition of the microbiome has a significant impact on host barrier permeability, specifically affecting the integrity of epithelial barriers in the gut and respiratory tract. Commensal microorganisms actively promote the expression of tight junction proteins and mucus production, which play a crucial role in maintaining barrier integrity and preventing the penetration of pathogens [148]. However, dysbiosis or alterations in the microbiome composition can compromise the function of these barriers, resulting in increased permeability and facilitating the entry of pathogens into host tissues [65,149]. Consequently, this heightened barrier permeability contributes to the development or worsening of infectious diseases.

The microbiome also exerts influence over host metabolic inflammation, particularly in conditions such as obesity and metabolic syndrome [150,151]. Dysbiosis observed in these conditions can lead to changes in the production of inflammatory mediators and adipokines, ultimately contributing to a pro-inflammatory state. This chronic, low-grade inflammation can have adverse effects on immune responses and create an environment that is conducive to the growth and persistence of pathogens [152]. Moreover, alterations in the composition of the gut microbiota can affect lipid metabolism and insulin sensitivity, further impacting an individual’s susceptibility to infectious diseases [153,154].

Certain changes in the composition of the microbiome have been associated with immunodeficiencies, which can manifest as deficiencies in specific immune cell populations or impaired immune responses [155,156,157]. Disruptions in the gut microbiota, for example, have been linked to reduced numbers of regulatory T cells and impaired T cell function, potentially resulting in increased vulnerability to infections [65,158,159]. Understanding these microbiome-associated immunodeficiencies is crucial for identifying individuals who may be at higher risk of developing infectious diseases and developing targeted interventions to restore immune function.

The microbiome has the ability to influence host stress responses by interacting with the neuroendocrine system and the hypothalamic–pituitary–adrenal (HPA) axis [160,161]. Commensal microorganisms can impact the production and signaling of stress hormones, which ultimately affect immune function and susceptibility to infections [162]. Furthermore, stress-induced alterations in the microbiome can exacerbate these effects, creating a feedback loop that may significantly impact infectious disease pathogenesis [163,164].

Commensal microorganisms are actively involved in breaking down and absorbing dietary components, thereby influencing the availability of metabolites that can impact host physiology and immune function [165,166]. Dysbiosis can lead to changes in metabolite absorption and availability, potentially affecting immune responses and an individual’s susceptibility to infections. For instance, alterations in microbial metabolism can influence the production of metabolites with immunomodulatory properties, thereby influencing host immune regulation and responses to pathogens [117,119].

**Figure 1:**
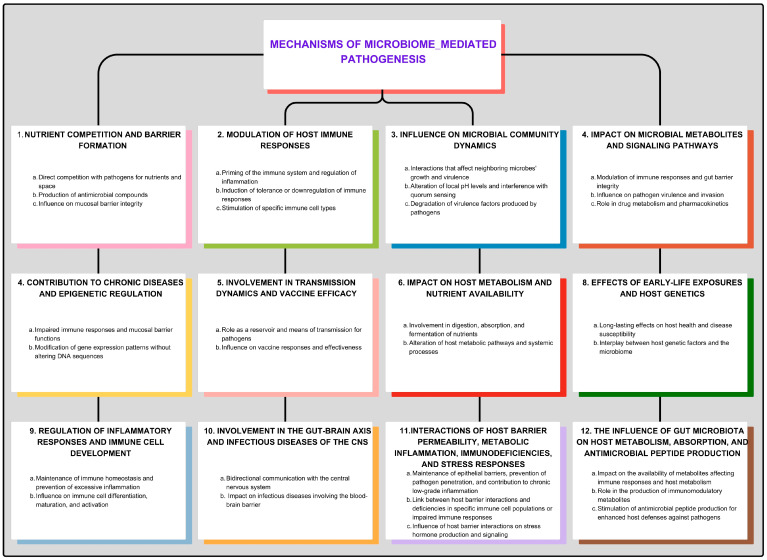
Mechanisms of microbiome-mediated pathogenesis. The diagram depicts the intricate relationship between a host organism and its microbial companions, detailing how this interplay can result in disease-causing scenarios. It sheds light on various ways through which the microbiome can influence the development of infectious diseases.

The microbiome has a significant influence on the production of antimicrobial peptides by host cells, which play a critical role in defending against pathogens. Commensal microorganisms actively stimulate the expression of genes that encode antimicrobial peptides, thereby enhancing host defenses against infections [167,168,169]. However, dysbiosis can lead to disruptions in antimicrobial peptide production, potentially compromising host immune responses and increasing susceptibility to infectious diseases. Some mechanisms of microbiome-mediated pathogenesis are illustrated in Figure 1.

## 4. Impact of Microbiome on Treatment Outcomes

The impact of the microbiome on treatment outcomes in infectious diseases has become a subject of increasing interest in the field of medical research. These microorganisms play a vital role in maintaining the overall health and balance of our body systems, particularly our immune system.

Recent studies have demonstrated that the composition and diversity of the microbiome can significantly influence the effectiveness of treatments for infectious diseases. For example, the use of antibiotics to treat bacterial infections can disrupt the balance of the microbiome by eliminating both harmful and beneficial bacteria [170,171]. This disruption can lead to the overgrowth of certain harmful bacteria, such as *C. difficile*, which can cause severe diarrhea and other complications.

Furthermore, the microbiome has been found to impact the response to antiviral therapies by impacting drug metabolism, immune system activation, drug resistance, inflammation, and offering potential for personalized medicine approaches [172,173,174]. Individuals with a specific composition of gut bacteria may have a higher likelihood of responding positively to antiviral medications for hepatitis B [175,176,177,178,179]. Similarly, the composition of the respiratory tract microbiome has been linked to the severity and duration of respiratory viral infections like influenza [180,181].

The microbiome can influence not only the effectiveness of treatment but also the development of drug resistance in infectious diseases [182]. Certain bacteria within the microbiome have been found to transfer antibiotic resistance genes to pathogenic bacteria, making them more difficult to treat [183,184]. This underscores the importance of understanding the role of the microbiome in treatment outcomes and developing strategies to reduce the emergence of drug resistance.

**Figure 2:**
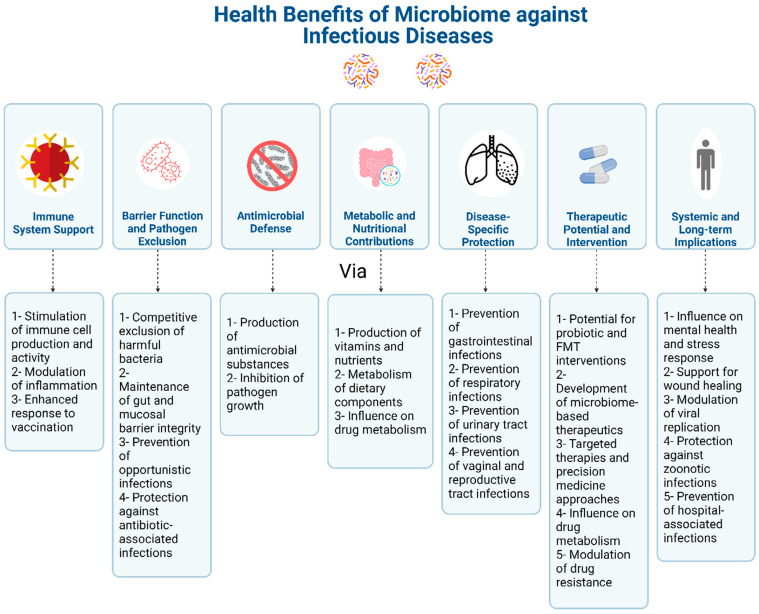
Health benefits of the microbiome against infectious diseases. This figure illustrates the various ways in which the microbiome contributes to protecting the human body against infectious diseases.

There is also evidence that influencing the microbiome can improve treatment outcomes for infectious diseases. This can be achieved through various approaches, including probiotics, i.e., live microorganisms that provide health benefits when consumed (Figure 2). Probiotics have shown promise in restoring microbial balance and improving response to treatment for conditions such as recurrent UTIs and antibiotic-associated diarrhea [185,186,187].

Another crucial aspect of the microbiome’s impact on treatment outcomes is its role in modulating host immune responses. The microbiome has been found to interact with the immune system, influencing its development, function, and ability to fight off infections. Imbalances in the microbiome, such as dysbiosis or alterations in microbial diversity, have been associated with an increased susceptibility to infections and a poor response to treatment. Understanding these interactions can help identify novel therapeutic targets to enhance immune responses and improve treatment outcomes [172,188]. Furthermore, the microbiome can influence the pharmacokinetics and pharmacodynamics of antimicrobial drugs [189]. Certain bacteria within the microbiome can metabolize or modify drugs, affecting their efficacy and toxicity. For example, gut bacteria can activate or deactivate specific antibiotics, potentially altering their therapeutic effect [190,191,192]. Additionally, the presence of particular bacteria in the gut can impact the absorption, distribution, and elimination of drugs, leading to variations in drug concentrations and responses [193,194]. Considering these microbiome–drug interactions can help optimize treatment regimens and minimize adverse effects.

The impact of the microbiome extends beyond bacterial infections and antiviral therapies. It also plays a role in fungal infections and antifungal treatments. The composition of the microbiome has been associated with susceptibility to fungal infections such as candidiasis and aspergillosis [195,196]. Moreover, some studies suggest that certain gut bacteria can enhance the antifungal activity of medications or inhibit the growth of pathogenic fungi. These findings highlight the potential for targeting the microbiome as an adjunctive therapy for fungal infections.

Recent studies have emphasized the role of the microbiome in shaping the effectiveness of immunotherapy in infectious diseases. Immunotherapy is a promising approach that utilizes the body’s immune system to combat infections. However, the efficacy of immunotherapy can vary among individuals, and the microbiome has emerged as a potential contributor to this variability [197]. Certain bacteria within the microbiome have been found to interact with immune cells and influence the response to immunotherapy [198]. Understanding the specific mechanisms by which the microbiome affects immunotherapy outcomes can help optimize treatment strategies and enhance patient outcomes.

Moreover, the impact of the microbiome on treatment outcomes extends beyond the individual level to population health. The composition of the microbiome can vary among different populations due to factors such as geography, diet, and lifestyle [199]. The impact of meat on the microbiota in a vegan diet is still uncertain, as factors such as origin, sex, age, and pre-existing illnesses contribute to the observed differences in the gut microbiota of long-term vegetarians and vegans compared with omnivores [182]. While interpersonal variability in enterotype composition has shown no significant effect of diet on the microbiota, extreme diets can cause temporary changes. High-fiber plant-based diets, including vegetarian, vegan, and Mediterranean diets, have been shown to have positive effects on health by promoting the growth of beneficial bacteria like *Rominococcus bromii* and reducing the Frimicutes/Bacteroidetes ratio [200,201,202,203]. However, different diets have varying impacts on the microbiome. Very low-calorie ketogenic diets, for example, significantly alter gut microbiota composition by promoting the growth of leanness-associated bacteria [204]. These variations can contribute to differences in susceptibility to infectious diseases and responses to treatments. By studying the microbiome at the population level, we can gain insights into the factors that influence treatment outcomes and develop targeted interventions to improve public health measures against infectious diseases.

One area of interest in studying the microbiome’s impact on treatment outcomes is antibiotic stewardship. Understanding the role of the microbiome in treatment outcomes can provide insights into strategies for optimizing antibiotic use [72,205]. By considering an individual’s microbiome composition, healthcare providers can make more informed decisions about selecting the most appropriate antibiotics, dosages, and treatment durations. This personalized approach can help minimize disruptions to the microbiome and reduce the risk of adverse outcomes, such as the development of antibiotic-resistant infections.

Furthermore, the microbiome’s impact on treatment outcomes extends beyond direct antimicrobial therapies to include the effectiveness of other therapeutic modalities, such as vaccines [206,207,208]. The presence of specific microbes in the gut can shape immune responses to vaccines [208], affecting their efficacy. By understanding these interactions, we can explore strategies to modulate the microbiome to enhance vaccine responses, potentially leading to improved protection against infectious diseases.

The microbiome has been implicated in the development of immune-related disorders and autoimmune diseases. Disruptions in the microbiome can lead to dysregulation of immune responses, resulting in chronic inflammation and increased susceptibility to infections [65]. Understanding the intricate interactions between the microbiome and the immune system can provide insights into novel therapeutic approaches. For example, fecal microbiota transplantation (FMT), which involves transferring healthy microbiota from a donor to a recipient, has shown promise in treating certain infections and immune-related conditions [209,210]. By restoring microbial balance, FMT can help modulate immune responses and improve treatment outcomes.

Moreover, the impact of the microbiome on treatment outcomes is not limited to individual patients. It also has implications for public health and infection control practices. The composition of the microbiome can influence the transmission and spread of infectious diseases within communities. For instance, certain strains of bacteria within the microbiome may enhance or inhibit the colonization and transmission of pathogens [211,212]. Understanding these dynamics can inform infection prevention strategies, such as the use of probiotics or targeted hygiene practices, to reduce the risk of disease transmission [89,213]. Additionally, studying the impact of the microbiome on treatment outcomes at a population level can help identify patterns and trends that guide public health interventions and resource allocation.

Recent technological advances have allowed for more comprehensive and precise analysis of the microbiome, leading to a deeper understanding of its impact on treatment outcomes in infectious diseases. High-throughput sequencing techniques, such as metagenomics, have enabled researchers to identify and characterize the vast array of microorganisms present in the human microbiome [214]. This has provided valuable insights into the microbial communities associated with different infectious diseases and their responses to treatment. Additionally, advancements in bioinformatics and computational tools have facilitated the analysis of large-scale microbiome datasets, allowing researchers to identify microbial signatures associated with treatment outcomes [215,216]. These technological advancements have accelerated our understanding of the microbiome’s role in infectious diseases and opened up new avenues for targeted therapeutic interventions.

Furthermore, the impact of the microbiome on treatment outcomes extends beyond infectious diseases alone. It also affects other areas of medicine, including cancer therapy. The microbiome has been implicated in modulating responses to cancer immunotherapy, influencing both treatment efficacy and toxicity. Specific gut bacteria have been associated with improved or reduced responses to immunotherapy drugs, highlighting the potential for manipulating the microbiome to enhance treatment outcomes in cancer patients [217,218]. This emerging field of research, known as microbial oncology, holds promise for revolutionizing cancer treatment strategies and improving patient outcomes [219,220,221].

One area of growing interest in this field is the development of microbiome-based therapeutics. Researchers are exploring the potential of utilizing specific microorganisms or their byproducts to target and treat infectious diseases. For example, certain beneficial bacteria found in the microbiome have been identified for their ability to produce antimicrobial substances that can inhibit the growth of pathogenic bacteria [222]. These antimicrobial-producing bacteria, known as probiotics, have shown promise in preventing or treating various infections [223]. Additionally, microbial-derived molecules, such as bacteriocins or phages, are being investigated as alternatives to traditional antibiotics. These microbiome-based therapeutics hold potential for providing targeted and tailored treatment options that minimize disruption to the overall microbiome while effectively combating infectious diseases.

Another area of research focus is understanding the impact of lifestyle factors on the microbiome and subsequent treatment outcomes in infectious diseases. It is now recognized that these lifestyle factors can affect an individual’s susceptibility to infections and response to treatments [224]. For instance, studies have linked a high-fiber diet to a more diverse and beneficial microbiome, which may enhance immune response and improve treatment outcomes [225,226]. Recognizing the role of lifestyle factors in shaping the microbiome opens up opportunities for interventions such as dietary modifications or stress reduction techniques that can complement traditional treatments and improve patient outcomes.

The study of the microbiome’s impact on treatment outcomes has revealed important insights into the concept of the “pathobiome”. This refers to the specific microbiome signatures associated with different diseases, allowing for targeted interventions and personalized treatment approaches. Researchers have identified distinct microbial communities linked to respiratory tract infections, UTIs, and skin infections [227,228]. Understanding these pathobiomes can guide therapeutic strategies by targeting specific microorganisms or manipulating the overall microbial composition to enhance treatment efficacy and improve patient outcomes.

Furthermore, the microbiome’s influence on treatment outcomes extends beyond infectious diseases to secondary health complications. Disruptions in the gut microbiome have been linked to an increased risk of developing conditions such as IBD, metabolic disorders, and mental health disorders [229,230]. These secondary health complications can further influence treatment outcomes for infectious diseases. Therefore, addressing the role of the microbiome in these comorbidities is crucial for a comprehensive understanding of treatment outcomes and developing holistic therapeutic approaches that consider the interplay between the microbiome and broader health conditions.

Moreover, the impact of the microbiome on treatment outcomes extends to chronic infectious diseases that require long-term management. In conditions such as HIV, hepatitis C, and tuberculosis, the microbiome has been found to influence disease progression, treatment response, and complications [231]. For instance, in individuals with HIV, the gut microbiome has been associated with immune activation and inflammation, impacting disease severity and response to antiretroviral therapy [232,233]. Similarly, in hepatitis C infection, the composition of the gut microbiome has been linked to treatment outcomes and liver disease progression. Understanding these interactions can inform personalized approaches to managing chronic infectious diseases and improving long-term treatment outcomes.

The microbiome’s impact on treatment outcomes is not limited to internal infections but also extends to skin microbiota and treatment outcomes in dermatological infectious conditions. The skin microbiome, which consists of a diverse community of microorganisms, plays a crucial role in maintaining skin health and protecting against infections. Disruptions in the skin microbiome have been associated with various dermatological infections such as acne, eczema, and fungal skin infections [234,235,236]. Understanding how the skin microbiome influences treatment outcomes can guide the development of targeted therapies that restore microbial balance and improve skin health [237,238]. Furthermore, considering the interplay between the skin microbiome and topical treatments can lead to more effective and personalized approaches for managing infectious skin conditions.

Additionally, the microbiome’s impact on treatment outcomes is relevant in the context of hospital-acquired infections. Factors such as antibiotic use, invasive procedures, and prolonged hospital stays can disrupt the microbiome of hospitalized patients, leading to imbalances and increased susceptibility to infections [239]. Understanding how these perturbations affect treatment outcomes is crucial for improving patient safety and reducing the risk of hospital-acquired infections [240]. Strategies aimed at preserving the microbiome during hospitalization, such as targeted antibiotic stewardship and infection prevention measures, can help mitigate the impact on treatment outcomes and reduce the burden of healthcare-associated infections.

The influence of the microbiome on treatment outcomes also extends to the pediatric population. The establishment of the infant microbiome during birth and early infancy has been linked to long-term health outcomes, including immune system development and disease susceptibility [241,242,243]. Disruptions in the early-life microbiome have been associated with an increased risk of infections and immune-related disorders in children. Understanding how early microbiome development influences treatment outcomes is essential for developing interventions that support healthy microbial colonization and improve resistance to infections in pediatric patients.

Moreover, the microbiome’s impact on treatment outcomes extends to antimicrobial resistance. Studies have shown that alterations in the composition and diversity of the microbiome can influence the emergence and spread of antimicrobial resistance [244,245]. Certain bacteria within the microbiome can harbor resistance genes and transfer them to pathogenic bacteria, contributing to the development of resistance [246]. Understanding how the microbiome influences antimicrobial resistance is essential for developing strategies to mitigate its impact. Interventions aimed at preserving microbial balance and minimizing disruptions to the microbiome during antibiotic treatments may help reduce the risk of promoting antimicrobial resistance.

In addition, the impact of the microbiome on treatment outcomes has implications for global health and infectious disease management in resource-limited settings. The composition and diversity of the microbiome can vary across different populations and geographic regions, influencing susceptibility to infections and treatment responses [247,248]. Recognizing these variations is crucial for developing tailored interventions that account for the unique microbiome profiles of diverse populations. Furthermore, understanding the impact of the microbiome on treatment outcomes in resource-limited settings can guide the development of cost-effective and sustainable strategies for managing infectious diseases in these contexts.

## 5. Innovative Treatment Strategies Targeting the Microbiome

**Figure 3:**
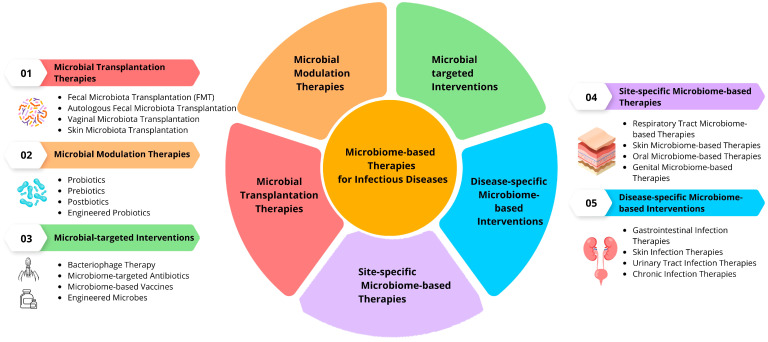
Different approaches of microbiome-based therapies for infectious diseases. This figure illustrates various strategies and approaches for utilizing the microbiome as a therapeutic intervention in the treatment of infectious diseases.

In recent years, there has been a growing recognition of the important role that the microbiome plays in maintaining our overall health [2]. The microbiome, which refers to the collection of microorganisms that inhabit our bodies, not only aids in digestion but also affects our immune system and protects us against infectious diseases. As we delve deeper into understanding the intricacies of the microbiome, researchers have begun to explore innovative treatment strategies that target these microbial communities to combat infectious diseases (Figure 3). One such strategy is the use of probiotics, which are live microorganisms that, when administered in adequate amounts, confer a health benefit on the host. Probiotics have shown promise in preventing and treating various infectious diseases by restoring the balance of beneficial bacteria in the microbiome. For example, studies have demonstrated that certain strains of probiotics can reduce the duration and severity of gastrointestinal infections caused by pathogens such as *C. difficile* and *Salmonella* [213,249].

Another innovative approach involves the use of FMT, a procedure where fecal matter from a healthy donor is transferred to a recipient with a disrupted microbiome. FMT has gained recognition as an effective treatment for recurrent *C. difficile* infection, with success rates exceeding 90%. This approach aims to restore a diverse and healthy microbiome in the recipient, thereby outcompeting and eliminating harmful pathogens [250,251].

Furthermore, researchers are investigating the potential of using phage therapy to target specific pathogenic bacteria within the microbiome. Bacteriophages are viruses that infect and kill bacteria, making them an attractive alternative to antibiotics [252,253]. By selectively targeting harmful bacteria while preserving beneficial ones, phage therapy has the potential to treat infectious diseases without disrupting the delicate balance of the microbiome.

An advance in sequencing technologies has allowed researchers to identify and characterize specific microbial signatures associated with different infectious diseases [254,255]. This knowledge opens up the possibility of developing personalized treatments that target the unique microbiota composition of each patient. By tailoring interventions to individual microbiomes, treatment efficacy could be significantly improved.

In addition to the aforementioned treatment strategies, researchers are also exploring the potential of microbiome-modulating drugs to combat infectious diseases [76,256]. These drugs aim to directly manipulate the composition and function of the microbiome to promote a healthy microbial community. For instance, small molecules known as postbiotics have emerged as a promising approach. Postbiotics are metabolic byproducts of probiotic bacteria that exhibit beneficial effects on the host [257,258,259]. They can modulate immune responses, enhance barrier function, and inhibit the growth of pathogens, making them attractive candidates for therapeutic interventions.

Furthermore, innovative technologies such as synthetic biology and genetic engineering are being utilized to engineer the microbiome for targeted disease treatment. Researchers are developing genetically modified probiotic bacteria that can produce specific antimicrobial peptides or molecules that enhance host immune responses [260,261]. These engineered probiotics have the potential to provide a more targeted and effective approach to combating infectious diseases by leveraging the natural abilities of these microorganisms.

Another area of active research is the investigation of the crosstalk between the microbiome and the immune system [262]. The microbiome has been shown to influence immune responses, and dysbiosis (an imbalance in the microbiome) has been associated with increased susceptibility to infections [90]. By understanding the intricate interactions between the microbiome and the immune system, researchers can develop novel strategies that modulate immune responses to enhance the body’s ability to fight off pathogens. Additionally, innovative diagnostic tools are being developed to assess the composition and function of the microbiome in real time. These tools, such as metagenomic sequencing and metabolomics, provide valuable insights into the dynamics of the microbiome during infectious diseases. By monitoring changes in the microbiome, clinicians can tailor treatment strategies and track the effectiveness of interventions, leading to more personalized and precise approaches to combating infectious diseases.

In recent years, there has been a growing interest in the potential of bacteriophage therapy as an innovative treatment strategy to combat infectious diseases [263,264]. Bacteriophages, also known as phages, are viruses that specifically target and infect bacteria. They have the ability to replicate within bacterial cells and ultimately cause their destruction. This targeted approach offers a potential alternative to traditional antibiotics, which can have broad-spectrum effects and lead to the development of antibiotic resistance.

Phage therapy involves the isolation and purification of specific phages that are known to target and kill the pathogenic bacteria causing the infection [265,266]. These phages are then administered to the patient, either orally, topically, or intravenously. The phages selectively replicate within the target bacteria, leading to their lysis and subsequent elimination from the body [267,268]. This approach has shown promising results in the treatment of various infectious diseases, including respiratory infections, UTIs, and skin infections.

Furthermore, researchers are exploring the potential of using engineered phages to enhance their therapeutic efficacy. By genetically modifying phages, scientists can improve their ability to recognize and infect specific bacterial strains, increasing their specificity and effectiveness. Additionally, engineered phages can be modified to carry payloads such as antimicrobial peptides or genes that encode antimicrobial substances, further enhancing their killing capacity.

Another innovative approach involves the use of microbiome-based therapeutics to target infectious diseases. The microbiome consists of trillions of microorganisms that inhabit various regions of our body, including the skin, gut, and respiratory tract. These microorganisms play a crucial role in maintaining health and preventing infections. Researchers are investigating the use of microbiome-based interventions, such as prebiotics and postbiotics, to promote a healthy microbiome and enhance the body’s natural defense mechanisms against pathogens [269,270].

Prebiotics are substances that selectively promote the growth of beneficial microorganisms in the gut. By providing a favorable environment for these beneficial bacteria to thrive, prebiotics can help restore microbial balance and strengthen the immune system [271,272,273]. Postbiotics, on the other hand, are the metabolic byproducts of beneficial bacteria that exhibit various health-promoting effects. These include antimicrobial activity against pathogens, modulation of immune responses, and enhancement of gut barrier function [274,275,276].

One approach is the use of nanotechnology to deliver antimicrobial agents directly to the site of infection [277,278,279]. Nano-sized particles can be engineered to encapsulate antimicrobial compounds and selectively target specific pathogens within the microbiome [280,281]. These nanoparticles can be designed to release the antimicrobial agents in a controlled manner, maximizing their effectiveness while minimizing off-target effects. This targeted delivery system holds great potential for enhancing the efficacy of antimicrobial treatments and reducing the development of resistance [280,282].

Another emerging strategy involves the modulation of the gut–brain axis to influence the microbiome and combat infectious diseases [283]. The gut–brain axis refers to the bidirectional communication between the gut and the brain, mediated by neural, endocrine, and immune pathways [284]. Studies have shown that disruptions in this axis can affect the composition and function of the microbiome, leading to increased susceptibility to infections [283]. By targeting the gut–brain axis through interventions such as dietary modifications, stress reduction techniques, or even neurostimulation, researchers aim to restore a healthy microbiome and enhance the body’s ability to fight off pathogens.

Furthermore, advancements in bioinformatics and computational modeling are playing a crucial role in understanding the complex interactions within the microbiome and guiding treatment strategies [285,286]. By analyzing large datasets of microbial genomic information, researchers can identify key microbial signatures associated with specific infections or disease states. This knowledge can then be used to develop predictive models that help predict disease progression, treatment response, and potential therapeutic targets within the microbiome.

Additionally, there is a growing interest in exploring the potential of microbiome transplantation from healthy individuals as a treatment strategy for infectious diseases. Similar to FMT, this approach involves transferring a diverse range of beneficial microorganisms from a healthy donor to an infected individual. By replenishing the microbial community with diverse and functional bacteria, this therapy aims to restore microbial balance and enhance the immune response against pathogens.

One emerging area of research is the development of microbiome-based vaccines. Traditional vaccines work by stimulating the immune system to recognize and mount a response against specific pathogens. Microbiome-based vaccines take advantage of the interactions between the microbiome and the immune system to develop novel vaccination approaches [287]. By targeting specific components of the microbiome that play a role in immune modulation, we aim to elicit a strong and protective immune response against pathogens [80,90]. These vaccines have the potential to provide long-lasting protection against infectious diseases while minimizing the risk of adverse reactions.

Another approach involves the use of antimicrobial peptides (AMPs) derived from the microbiome [288,289]. AMPs are small proteins produced by various organisms, including bacteria, fungi, and even our own cells [290]. They possess broad-spectrum antimicrobial activity and can effectively kill or inhibit the growth of pathogens. Researchers are exploring the use of synthetic or naturally occurring AMPs as therapeutics to combat infectious diseases [291]. These peptides can be administered topically, orally, or through targeted delivery systems to specifically target the site of infection while minimizing damage to beneficial microbes [291,292].

Furthermore, there is a growing interest in utilizing microbiome-targeted therapies in combination with traditional antibiotics. Antibiotics are essential for treating many infectious diseases; however, their broad-spectrum nature can disrupt the delicate balance of the microbiome and contribute to antibiotic resistance [171,293]. Researchers are investigating strategies to selectively target pathogens while preserving the diversity and functionality of the microbiome. This includes developing antibiotics that specifically target pathogenic bacteria or using adjuvants, such as probiotics or prebiotics, alongside antibiotics to mitigate their negative effects on the microbiome.

Additionally, researchers are exploring the potential of using CRISPR-Cas systems to selectively eliminate or modify harmful bacteria within the microbiome [294]. CRISPR-Cas is a revolutionary gene-editing technology that allows precise modifications of genetic material [295,296]. By utilizing CRISPR-Cas systems, we can design specific guide RNAs to target and destroy pathogenic bacteria or disrupt their virulence factors. This approach holds promise for developing highly targeted and customizable treatments for infectious diseases.

One promising area of research is the development of microbiome-targeted nanoparticles for drug delivery [297,298]. These nanoparticles can be engineered to specifically target and penetrate the microbial communities within the body. By encapsulating antimicrobial agents or other therapeutic compounds, these nanoparticles can deliver the treatment directly to the site of infection, increasing its effectiveness and reducing systemic side effects [298,299,300,301,302,303]. Additionally, the nanoparticles can be designed to release the therapeutic agents in response to specific cues or conditions within the microbiome, further enhancing their targeted action.

Another emerging approach involves utilizing gene therapy techniques to modulate the microbiome and combat infectious diseases. By introducing specific genes into the microbiome, researchers aim to enhance the production of antimicrobial compounds, boost immune responses, or restore microbial balance. This approach holds potential for developing targeted and long-lasting interventions that can help prevent or treat infectious diseases.

In recent years, there has been a growing interest in the potential of microbial-derived products as therapeutic agents. These products include metabolites, enzymes, and proteins produced by the microbiome that have antimicrobial or immunomodulatory properties [304,305,306,307,308]. Researchers are exploring ways to harness these natural products for therapeutic purposes. For example, certain microbial metabolites have shown antimicrobial activity against drug-resistant pathogens, making them attractive candidates for developing new treatments. Furthermore, enzymes produced by the microbiome can be used to degrade biofilms formed by pathogenic bacteria, enhancing the effectiveness of antimicrobial therapies.

Additionally, researchers are investigating the potential of bioengineered microbiomes as a treatment strategy. By engineering synthetic microbial communities with specific functional characteristics, scientists aim to create microbiomes that can outcompete and eliminate pathogenic bacteria [309,310]. These bioengineered microbiomes can be designed to produce antimicrobial substances, enhance immune responses, or restore microbial diversity and stability. This approach offers a unique opportunity to tailor the microbiome to combat specific infectious diseases.

One emerging area of research is the use of bacteriocins as therapeutic agents. Bacteriocins are AMPs produced by bacteria that can selectively target and kill other bacteria, including pathogenic strains [311,312]. The potential of harnessing these naturally occurring antimicrobial compounds for the treatment of infectious diseases is being explored. Bacteriocins can be engineered or modified to enhance their stability, potency, and specificity against target pathogens [313,314]. By leveraging bacteriocins, we aim to develop targeted antimicrobial therapies that minimize harm to the beneficial microbes within the microbiome.

Another approach involves the use of microbiome transplantation from healthy animal models to treat infectious diseases. Animal models with a naturally resistant or tolerant microbiome can serve as sources for microbiome transplantation to infected or susceptible individuals [315]. By transferring the microbiota from these resistant animals, we aim to enhance the microbial diversity and functionality of the recipient’s microbiome, providing a more robust defense against pathogens. This approach holds promise for developing novel treatments, particularly for infections that are difficult to treat with conventional therapies.

Furthermore, there is a growing interest in exploring the potential of host-directed therapies that modulate the host immune response to combat infectious diseases. The microbiome has been shown to play a crucial role in regulating immune function, and dysbiosis can contribute to immune dysfunction and increased susceptibility to infections [90,316]. Exploring interventions such as immune-modulating drugs, dietary interventions, or fecal transplantation to restore a healthy balance in the microbiome and enhance host immunity against pathogens is very crucial.

Additionally, researchers are investigating the potential of using synthetic biology approaches to engineer microbiomes for targeted disease treatment [317,318]. By designing and introducing synthetic genetic circuits into specific microbial populations within the microbiome, we aim to enhance their ability to produce antimicrobial compounds, compete with pathogenic strains, or modulate immune responses. This field of research offers exciting possibilities for developing tailored therapies that can precisely target and manipulate the microbiome to combat infectious diseases.

## 6. Personalized Medicine Approaches

Personalized medicine is an innovative approach that customizes medical treatments based on individual characteristics, including genetics, lifestyle, and environment. Its primary goal is to enhance effectiveness by offering accurate diagnoses, predicting disease risks, and selecting tailored therapies. By optimizing healthcare outcomes and improving patient well-being, personalized medicine strives to revolutionize the field of healthcare. Personalized medicine has the potential to revolutionize the approach to infectious diseases by analyzing an individual’s microbiome profile [319]. The microbiome refers to the vast community of microorganisms that reside within and on our bodies, playing a crucial role in maintaining our health. Recent advancements in DNA sequencing technologies have made it possible to characterize the microbiome in unprecedented detail, providing valuable insights into infectious diseases [320]. The composition of an individual’s microbiome has been linked to various infectious diseases, including respiratory infections, gastrointestinal infections, and STIs [17,24,80]. By studying the microbial communities associated with these diseases, we can identify potential pathogens and their interactions with the host immune system. This knowledge can guide the development of targeted therapies that specifically address the underlying microbial imbalances associated with the disease.

Furthermore, the microbiome has been shown to influence the efficacy and toxicity of antimicrobial agents. Different microorganisms within the microbiome possess varying degrees of resistance or susceptibility to specific drugs. By analyzing an individual’s microbiome profile, healthcare professionals can predict drug responses and select appropriate antibiotics or antivirals that are more likely to be effective against the specific pathogens present [321,322]. This personalized approach can minimize the risk of treatment failure or adverse drug reactions, leading to improved therapeutic outcomes.

In addition to guiding treatment decisions, understanding an individual’s microbiome can also inform preventive strategies for infectious diseases. By identifying individuals who are more susceptible to certain infections based on their microbiome composition, targeted interventions such as probiotic supplementation or prebiotic dietary modifications can be implemented to restore microbial balance and reduce the risk of infection.

Moreover, the microbiome plays a critical role in modulating the immune system, which is essential for fighting off infections. By understanding an individual’s microbiome profile, healthcare professionals can gain insights into the functioning of the immune system and its dysregulation in infectious diseases [323,324,325]. This knowledge can guide the development of immunomodulatory therapies that target specific pathways or immune cell populations, ultimately enhancing the body’s ability to combat infections.

Personalized medicine based on the microbiome also holds promise in the field of diagnostics. By analyzing an individual’s microbiome, it becomes possible to identify patterns or signatures associated with different infections [326,327]. These microbial biomarkers can serve as diagnostic tools for the early detection and monitoring of infectious diseases [328,329]. Researchers are investigating gut microbiota signatures, such as short-chain fatty acids (SCFAs), branched-chain amino acids (BCAAs), and trimethylamine N-oxide (TMAO), as potential biomarkers for infectious diseases [328]. Furthermore, associations between microbial diversity, gene expression patterns in the gut microbiota, and susceptibility to infectious diseases have also been observed [330]. Investigating microbiota signatures associated with invasive Candida infections offers insights into diagnostic and therapeutic targets [331]. Furthermore, the use of microbiome-based diagnostics can facilitate rapid and accurate identification of drug-resistant pathogens, enabling targeted antimicrobial therapy and preventing the spread of antibiotic resistance.

In the prevention of healthcare-associated infections (HAIs), personalized medicine approaches based on the microbiome can help identify patients who are at a higher risk of developing these infections. This knowledge can be used to implement targeted interventions, such as probiotic therapy or the use of prebiotics, to promote a healthy microbiome and reduce the risk of HAIs. Personalized medicine approaches that focus on the microbiome have the potential to greatly improve infection control practices in healthcare settings and enhance patient safety.

By understanding an individual’s microbiome composition, healthcare professionals can identify specific microbial species or functional pathways that may enhance or hinder treatment responses [317,332,333]. This knowledge can guide the development of strategies to modulate the microbiome, such as targeted probiotics or FMT, with the aim of improving therapeutic outcomes.

In terms of vaccine development, analyzing an individual’s microbiome can provide insights into the interactions between the immune system and resident microbial communities. This knowledge can guide the development of novel vaccines that harness the immune-modulating properties of the microbiome to enhance vaccine efficacy and duration of protection [106,108,334,335]. Microbiome-based vaccines have the potential to offer a more personalized and effective approach to preventing infectious diseases.

Furthermore, personalized medicine approaches based on an individual’s microbiome profile can contribute to precision antimicrobial therapy. By analyzing an individual’s microbiome profile, healthcare providers can identify the presence of antibiotic-resistant bacteria and predict their potential impact on treatment outcomes. This information can guide the selection of appropriate antibiotics or alternative antimicrobial strategies that are more likely to be effective against specific pathogens present, while minimizing the risk of promoting further antibiotic resistance.

The integration of big data technologies in personalized medicine offers exciting prospects for infectious diseases [335,336]. By combining vast amounts of microbiome data with clinical information and outcomes, researchers can identify patterns and correlations that may not be apparent through traditional methods [337,338,339]. AI algorithms can analyze these complex datasets and generate predictive models to guide personalized medicine interventions. This data-driven approach has the potential to revolutionize infectious disease management by providing clinicians with actionable insights for diagnosis, treatment selection, and monitoring of therapeutic responses.

Personalized medicine based on an individual’s microbiome profile also offers the opportunity to uncover new therapeutic targets for infectious diseases. The microbiome is a rich source of bioactive molecules and metabolites that can have diverse effects on human health and disease [323,324,340]. By studying the microbial communities within the microbiome, researchers can identify specific molecules or pathways that contribute to disease progression or protection against infections. This knowledge can guide the development of novel therapies that target these specific microbial components, potentially leading to more effective and tailored treatments for infectious diseases.

Moreover, personalized medicine in infectious diseases can extend beyond individual treatment to population-level interventions. By analyzing large-scale microbiome data from different populations, researchers can identify patterns and trends that may influence disease prevalence, transmission, and response to interventions. This information can guide public health strategies, such as vaccination campaigns or targeted antimicrobial stewardship programs, to address specific infectious diseases in different populations. Personalized medicine approaches that consider the microbiome have the potential to inform proactive measures for disease prevention and improve population-level health outcomes.

One emerging area of personalized medicine in infectious diseases is the role of the microbiome in predicting disease outcomes and complications [341]. By analyzing an individual’s microbiome profile, healthcare professionals can identify microbial signatures or biomarkers that are indicative of disease progression or the likelihood of developing complications, helping guide treatment decisions, facilitate early intervention, and improve patient management strategies.

Furthermore, personalized medicine based on an individual’s microbiome profile holds great potential for the development of novel therapeutics, such as microbiome-based interventions. By analyzing an individual’s microbiome profile, healthcare providers can identify specific microbial imbalances and design tailored interventions to restore a healthy microbiome and improve outcomes in infectious diseases. This can be achieved through targeted probiotic therapy, prebiotic supplementation, or even the development of microbial-based therapeutics. Personalized medicine based on an individual’s microbiome profile shows promise in identifying novel therapeutic targets for infectious diseases. By studying an individual’s microbiome profile, we can identify unique microbial species or functional pathways associated with specific infectious diseases, opening up new avenues for targeted therapies. Additionally, personalized medicine based on an individual’s microbiome profile can contribute to strategies for disease prevention and public health interventions by analyzing large-scale microbiome data from different populations to identify patterns and trends associated with disease susceptibility or transmission. This information can inform public health initiatives, such as vaccination campaigns or targeted antimicrobial stewardship programs.

## 7. Microbiome Challenges in Infectious Diseases

The study of the microbiome in the context of infectious diseases presents several challenges that we must overcome (Table 2). First, the microbiome itself is incredibly complex, consisting of a vast community of microorganisms, including bacteria, viruses, fungi, and other organisms [342]. Understanding the interactions between these microorganisms and their host in the context of infectious diseases is difficult due to this complexity. Second, the dynamic nature of the microbiome adds another layer of challenge, as its composition and diversity can vary significantly between individuals and over time [343]. This variability makes it challenging to establish consistent associations between specific microbial communities and infectious diseases. Third, studying the microbiome in infectious diseases requires advanced analytical tools and techniques [344,345,346]. Traditional culture-based methods are limited in their ability to identify and characterize the majority of microorganisms present in the microbiome. High-throughput sequencing technologies, such as metagenomic sequencing, are used to analyze microbial DNA but generate large amounts of data that require sophisticated bioinformatics analyses [347]. Fourth, ethical considerations play a crucial role in studying the microbiome. Accessing and analyzing samples raises privacy concerns, requiring researchers to navigate complex ethical frameworks to ensure informed consent, privacy protection, and proper communication of research findings. Lastly, the presence of unculturable or difficult-to-study microorganisms in the microbiome restricts our ability to fully characterize and understand their functional roles in infectious diseases [348].

The microbiome, which extends beyond the human body to include the environment, plays a role in the spread and transmission of infectious diseases. However, studying the microbiome within a broader ecological context adds complexity and requires interdisciplinary collaborations and data integration. Understanding the microbiome’s association with infectious diseases is further complicated by confounding factors such as host genetics, immune status, and underlying health conditions [24,349]. These factors must be carefully addressed through experimental design and statistical analysis to accurately identify microbial signatures and develop targeted interventions.

Translating microbiome research into clinical applications is challenging due to individual variations in the microbiome and evolving regulatory frameworks [350]. Establishing causality and developing effective interventions remain complex tasks, requiring further research, clinical trials, and collaboration between researchers, clinicians, and regulatory bodies. Standardization of methodologies and protocols is another challenge in microbiome research, hindering comparability and reproducibility. Efforts are being made to establish standardized protocols and reference datasets to overcome this obstacle [351,352,353].

While bacterial communities have received significant attention, the role of viruses, fungi, and other microorganisms in the microbiome remains less understood [354]. Investigating these components presents challenges such as limited knowledge of their interactions with the host immune system and difficulties in distinguishing between pathogenic and commensal microorganisms. Longitudinal studies tracking changes in the microbiome over time are necessary to understand temporal dynamics and causal relationships with infectious diseases [324,355]. However, conducting such studies poses logistical challenges related to follow-up, sample collection, data analysis, participant retention, compliance, and the potential impacts of interventions.

The translation of microbiome research into clinical practice faces hurdles, including regulatory barriers, and the need for robust clinical validation. Integration of microbiome-based approaches into healthcare systems requires collaborations between researchers, clinicians, industry partners, and regulatory agencies to establish evidence-based guidelines and ensure clinical utility and safety.

Careful consideration of factors like age, sex, geographical location, diet, medications, and underlying health conditions during study design and statistical analysis is essential for accurate findings [356,357,358,359,360]. Additionally, the vast amount of data generated from microbiome studies poses challenges in terms of storage, management, and analysis. Robust computational infrastructure and bioinformatics expertise are required to process and integrate such data effectively. The availability of computational resources can be a challenge for many research groups.

There is a need for deeper comprehension of the biological activities and interactions within the microbiome, which requires specialized techniques like metatranscriptomics and metabolomics [361]. Incorporating these techniques into routine microbiome research is challenging due to the need for expertise and resources. Interdisciplinary collaborations are necessary to study the microbiome in the context of infectious diseases effectively. Researchers from various fields, including microbiology, immunology, genetics, bioinformatics, and clinical medicine, need to work together to tackle the complexity of the microbiome [362]. However, building effective collaborations and fostering communication across disciplines can be challenging due to differences in terminology, methodologies, and research cultures. The heterogeneity within infectious diseases themselves poses a significant challenge. Different diseases have distinct etiologies, pathogenic mechanisms, and host responses, leading to variations in microbial factors contributing to each disease. Additionally, studying the microbiome across different stages of infection and disease progression adds another layer of complexity as the microbial communities may change dynamically over time [363]. Establishing causality between the microbiome and infectious diseases is another challenge. While associations between specific microbial communities and disease states have been identified, determining cause-and-effect relationships is challenging [364]. Experimental models can provide insights into the mechanisms by which the microbiome influences infectious diseases, but validating these findings in human populations requires careful integration of multiple lines of evidence. Standardization of sample collection and processing methods is crucial for cross-study comparisons and meta-analyses [365]. Variability in these protocols can introduce biases and hinder the reproducibility of the results. Efforts are being made to establish guidelines and best practices for sample collection and processing to ensure consistency and comparability across studies. Understanding the ecological dynamics within the microbiome and how perturbations impact its stability and function is essential. Factors like antibiotic usage, infection treatments, or changes in environmental conditions can disrupt the balance of the microbiome, leading to dysbiosis [366,367,368]. Determining the resilience of the microbiome and its capacity to recover from perturbations is crucial for developing targeted interventions. Data integration and interpretation pose challenges due to variations in study design, sampling protocols, sequencing platforms, and bioinformatics pipelines. Standardized data repositories and analytical frameworks are being developed to facilitate data sharing and integration. Microbial interdependencies and interactions within the microbiome add complexity. Understanding these intricate microbial networks and their dynamics is crucial for comprehending the overall functioning of the microbiome in infectious diseases [369,370]. Conducting large-scale, multicenter studies poses logistical and coordination challenges. Collaborative efforts are necessary to collect diverse samples from different populations, locations, and disease contexts [371]. Implementing standardized protocols, quality control measures, and coordinating data management require effective communication, resources, and infrastructure. Translating microbiome research into clinical practice and public health interventions requires rigorous validation, regulatory approvals, and evidence-based guidelines [372]. Overcoming barriers related to cost-effectiveness, accessibility, and feasibility is essential. Longitudinal studies with well-defined cohorts are needed to understand the temporal dynamics of the microbiome in infectious diseases. Long-term commitment, extensive follow-up, and careful coordination are required for sample collection at specific time points [373]. Standardized metadata collection and reporting are necessary to integrate and compare data across studies. Establishing standardized metadata standards and guidelines would facilitate data harmonization, improve reproducibility, and enable meta-analyses [373]. Identifying causal mechanisms between specific microbes or microbial functions and infectious diseases remains complex. Experimental validation and functional studies are required to establish causality. Exploring the “microbial dark matter” within the microbiome presents technological and conceptual challenges [374]. Innovative techniques can help shed light on uncultured microorganisms’ functions and interactions with the host [375,376]. Host genetics can shape the microbiome and impact susceptibility to infectious diseases. Comprehensive genetic studies and integrative analyses are essential for understanding the interplay between host genetics, the microbiome, and infectious diseases [377]. Early-life exposures can have long-term implications for the development of the microbiome and infectious disease susceptibility [378,379]. Longitudinal cohort studies are needed to study these effects into adulthood. The role of the microbiome in modulating immune responses during infectious diseases is an active area of investigation. Understanding this complex crosstalk requires comprehensive knowledge of immunology, microbiology, and host–microbe interactions.

The translation of microbiome research findings into actionable clinical interventions and public health strategies presents a major challenge. While there is growing evidence for the role of the microbiome in infectious diseases [380], implementing microbiome-based diagnostics, therapeutics, and preventive measures in clinical and public health settings requires robust evidence of efficacy, safety, and cost-effectiveness. Developing evidence-based guidelines, regulatory pathways, and healthcare policies to support the integration of microbiome-based approaches into routine clinical practice is essential for realizing the potential benefits of microbiome research in improving infectious disease management.

Certainly, another significant challenge in the study of the microbiome in the context of infectious diseases is the influence of environmental factors on microbial communities and disease outcomes. Environmental exposures, such as air pollution, water quality, urbanization, and occupational settings, can impact the microbiome and contribute to the spread and transmission of infectious diseases [97,381]. Understanding the complex interplay between environmental factors, the microbiome, and infectious diseases requires interdisciplinary research that integrates environmental science, epidemiology, and microbiome studies. Investigating how environmental exposures shape the microbial ecology and influence disease susceptibility is crucial for developing targeted interventions and public health strategies.

The impact of antibiotic resistance and usage on the microbiome is a critical challenge, as it can disrupt microbial communities and lead to negative health consequences [382]. The widespread use of antibiotics in various settings raises concerns about the emergence of resistance and its implications for infectious diseases. To mitigate these risks, studying the effects of antimicrobial agents on the microbiome and understanding microbial responses to selective pressures are crucial. Exploring the role of the microbiome in vaccine responses and efficacy is an emerging area of interest. The microbiome can influence individual responses to vaccination by modulating immune system development and function [93]. Understanding how specific microbial communities or functions impact vaccine-induced immune responses requires comprehensive studies. Integrating microbiome analyses into vaccine research can optimize vaccination strategies and improve effectiveness against infectious diseases. Addressing disparities in microbiome research and ensuring inclusivity across diverse populations is a critical challenge. Many studies have focused on high-income countries, leading to gaps in understanding microbiome diversity and function in different ethnic groups, regions, and socioeconomic settings. Diversifying study cohorts, promoting global collaborations, and ensuring equitable access to research opportunities are essential for advancing our understanding of the microbiome’s role in infectious diseases. Functional redundancy and plasticity within microbial communities pose challenges in identifying specific microbial contributors to infectious diseases. Different species can perform similar functions, making it difficult to pinpoint their roles. Understanding functional potential and plasticity requires advanced computational modeling and experimental approaches to unravel their implications for infectious diseases. The microbiome extends beyond the human body to other animal reservoirs and environmental sources. The virome, consisting of viruses in the human body and environment, presents unique challenges in microbiome research [383]. Characterizing the virome and understanding its role in infectious diseases require specialized techniques for viral detection and analysis. Advancements in sequencing, bioinformatics, and viral–host interactions are necessary to unravel the complex interplay between the virome and infectious diseases [383]. Ethical considerations related to microbiome research, data sharing, and privacy present ongoing challenges. Ensuring informed consent, privacy protection, and responsible data sharing practices are essential. Clear guidelines for sample collection, data management, and communication of research findings are necessary. Ethical frameworks for global collaborations and cross-cultural studies are crucial for promoting ethical standards. Diet plays a crucial role in shaping the microbiome, and alterations can influence disease susceptibility [384,385]. Understanding the relationship between diet, the microbiome, and infectious diseases requires comprehensive assessments and integrative analyses [386]. Emerging infectious diseases and their impact on the microbiome require international collaboration and surveillance efforts. Understanding the role of the microbiome in disease emergence necessitates coordinated efforts across geographic regions. Personalized approaches to studying the microbiome and infectious diseases are challenging due to individual variability [319]. Implementing personalized medicine strategies requires innovative analytical tools and models that accommodate diverse datasets [387]. Equitable access to research opportunities, resources, and interventions is an ongoing challenge. Promoting diversity in study cohorts, fostering inclusive research environments, and engaging under-represented communities are necessary. Lifestyle factors and socio-economic determinants impact the microbiome and disease outcomes. Understanding these interplays requires interdisciplinary research [388,389]. Chronic diseases and comorbidities can alter the microbiome and influence disease susceptibility. Investigating these relationships requires comprehensive studies [390]. Urbanization, globalization, and environmental changes impact the microbiome and disease dynamics. Integrated approaches are necessary to address these challenges. Interdisciplinary collaborations and knowledge translation are vital for addressing multifaceted challenges in microbiome research. Emerging technologies and data science revolutionize microbiome research but present challenges in data integration, standardization, and interpretation. Immune system aging influences the microbiome and its interactions with infectious agents [80,316]. Comprehensive immunological studies are required to understand these relationships. Microbial toxins, metabolites, and virulence factors impact host–microbe interactions [119]. Investigating their functional implications requires sophisticated studies. Robust data reproducibility, transparency, and rigor are essential in microbiome research. Standardized protocols, validation of analytical pipelines, and open access promote reliability. Understanding microbial interactions and community dynamics is crucial for disease outcomes. Advanced modeling and experimental studies are necessary to capture this complexity [391,392].

Addressing the impact of microbiome-derived immune modulation on infectious diseases presents a multifaceted challenge, as the microbiome has been implicated in modulating host immune responses, influencing immune tolerance, and shaping overall immune system development [90]. Understanding how microbial communities interact with the host immune system, regulate inflammatory processes, and influence immune responses to infectious agents requires comprehensive immunological studies, integrative analyses of host–microbe immune interactions, and a deeper understanding of the immunomodulatory effects of specific microbial taxa and functions. Furthermore, investigating the complex relationships between microbiome-derived immune modulation and infectious diseases is essential for developing targeted interventions that harness the immunomodulatory potential of beneficial microbes while mitigating the impacts of dysbiotic microbial communities [90]. Another significant challenge lies in addressing the influence of microbial adaptation and evolution on infectious diseases, as microbial communities within the microbiome can adapt to environmental changes, host immune responses, and therapeutic interventions, leading to the emergence of resistant or virulent strains that can impact disease outcomes [393,394,395]. This necessitates integrated approaches that consider microbial genomics, evolutionary biology, and population dynamics to understand how microbial adaptation and evolution influence infectious diseases [396].

Equally important is ensuring effective communication and knowledge dissemination across diverse stakeholders in microbiome research and infectious diseases to bridge the gap between researchers, clinicians, policymakers, industry partners, and the public [397]. This requires effective communication strategies, knowledge translation efforts, and public engagement initiatives that promote understanding and awareness of microbiome research findings and their implications for infectious diseases. Additionally, addressing the impact of microbial dysbiosis and ecological disruptions on disease susceptibility and progression is crucial, as dysbiosis has been associated with various infectious diseases and inflammatory conditions [24]. Understanding how dysbiosis and ecological disruptions within the microbiome influence disease states requires comprehensive ecological studies, advanced statistical methodologies, and a deeper understanding of microbial community stability and resilience.

Moreover, it is essential to address the impact of microbial co-infections and polymicrobial interactions on infectious diseases, as microbial communities within the microbiome can harbor diverse microorganisms that may interact synergistically or antagonistically, influencing disease outcomes [398,399]. This necessitates integrative multi-omic analyses, ecological modeling of microbial communities, and experimental studies to understand how microbial co-infections and polymicrobial interactions influence disease pathogenesis. Furthermore, addressing the influence of the microbiome on antimicrobial therapy and drug resistance is critical, as microbial communities within the microbiome can impact the efficacy of antimicrobial therapies, contribute to the emergence of resistance, and influence treatment outcomes. This requires integrated approaches that consider microbial genomics, pharmacology, and clinical studies to understand how the microbiome influences antimicrobial therapy responses and drug resistance.

Developing evidence-based policies, regulatory guidelines, and ethical frameworks that promote safe and responsible utilization of microbiome-based interventions and technologies is an ongoing challenge that necessitates collaboration between researchers, policymakers, industry partners, and regulatory agencies [400]. Additionally, addressing the influence of the microbiome on host metabolism and metabolic disorders is crucial, as the microbiome plays a crucial role in modulating host metabolism with potential implications for metabolic diseases such as diabetes, obesity, and cardiovascular conditions. Moreover, addressing the impact of the microbiome on neuroimmune interactions and neurological disorders is essential, as the microbiome has been implicated in influencing neuroimmune responses, neuroinflammation, and neurological conditions such as neurodegenerative diseases and psychiatric disorders [401].

Additionally, investigating the influence of microbiome-derived metabolites and signaling molecules on host physiology and disease outcomes is crucial for developing targeted interventions that leverage beneficial microbial metabolites while mitigating the impacts of dysbiotic microbial activities on host health [402]. Ensuring equitable access to microbiome-based interventions and healthcare services for vulnerable populations is an ongoing challenge that involves fostering diversity in study cohorts, promoting inclusive research environments, and developing outreach programs that engage under-represented communities.

One additional challenge is the need to understand the impact of the microbiome on immune development and immune-related disorders. The microbiome plays a crucial role in shaping the development and function of the immune system, with potential implications for allergies, autoimmune conditions, and immunodeficiencies. To understand these complex interactions, comprehensive immunological studies, integrative multi-omic analyses, and a deeper understanding of specific microbial communities’ immunomodulatory functions are required [90,403]. Furthermore, it is essential to address the influence of the microbiome on mucosal barrier function and mucosal disorders. The microbiome plays a crucial role in maintaining mucosal barrier integrity, regulating immune responses at mucosal surfaces, and influencing mucosal disorders such as IBD and mucosal infections. To gain insight into these relationships, comprehensive mucosal barrier studies, integrative analyses of host–microbe–mucosal interactions, and a deeper understanding of specific microbial taxa and functions’ mucosal functions are required.

Additionally, effective public health strategies that leverage microbiome research for disease prevention and intervention present an ongoing challenge. Developing evidence-based public health policies, preventive measures, and clinical guidelines that incorporate microbiome-based approaches requires collaboration between researchers, clinicians, policymakers, industry partners, and public health agencies. Addressing legal and ethical considerations related to microbiome-based interventions, data privacy, and clinical translation is crucial for responsible advancement in this field.

Another challenge is addressing the impact of the microbiome on reproductive health and reproductive disorders. The microbiome has been found to influence reproductive physiology, fertility, and reproductive disorders such as infertility and pregnancy complications [404,405,406]. To understand these interactions, comprehensive reproductive health studies, integrative analyses of host–microbe–reproductive interactions, and a deeper understanding of specific microbial taxa and functions’ reproductive functions are necessary.

Moreover, understanding the impact of the microbiome on respiratory health and respiratory disorders is crucial. The microbiome has been implicated in influencing respiratory physiology, immune responses, and respiratory disorders such as asthma, COPD, and respiratory infections [407]. To comprehend these relationships, comprehensive respiratory health studies, integrative analyses of host–microbe–respiratory interactions, and a deeper understanding of specific microbial taxa and functions’ respiratory functions are necessary.

Similarly, addressing the impact of the microbiome on skin health and dermatological disorders presents a multifaceted challenge. The skin microbiome plays a critical role in maintaining skin barrier function, regulating immune responses in the skin, and influencing dermatological conditions such as eczema, acne, and skin infections [408,409]. To gain insight into these interactions, comprehensive dermatological studies, integrative analyses of host–microbe–skin interactions, and a deeper understanding of specific microbial taxa and functions’ skin-related functions are required.

Additionally, addressing the influence of the microbiome on metabolic health and metabolic disorders is critical. The microbiome has been implicated in influencing host metabolism, nutrient processing, and energy homeostasis, potentially impacting metabolic diseases such as diabetes, obesity, and cardiovascular conditions [410]. To understand these complex interactions, comprehensive metabolic studies, integrative multi-omic analyses, and a deeper understanding of specific microbial communities’ metabolic functions are necessary.

Ensuring equitable access to microbiome-based interventions and healthcare services for vulnerable populations is another ongoing challenge. This involves addressing disparities in access to research opportunities, resources, and interventions by fostering diversity in study cohorts, promoting inclusive research environments, and developing outreach programs that engage under-represented communities. It also requires addressing social determinants of health, advocating for health equity policies, and building partnerships with community organizations to ensure that microbiome research benefits all populations.

Furthermore, it is crucial to understand the impact of the microbiome on oral health and oral disorders. The oral microbiome plays a crucial role in maintaining oral health, influencing immune responses in the oral cavity, and contributing to oral conditions such as periodontal disease, dental caries, and oral infections [411]. To gain insight into these relationships, comprehensive studies of oral health, integrative analyses of host–microbe-oral interactions, and a deeper understanding of specific microbial taxa and functions’ oral-related functions are necessary.

Moreover, addressing the influence of the microbiome on gastrointestinal health and gastrointestinal disorders presents a multifaceted challenge. The gut microbiome plays a crucial role in maintaining gut barrier function, regulating immune responses in the gastrointestinal tract, and influencing gastrointestinal conditions such as IBD, irritable bowel syndrome, and gastrointestinal infections [412,413]. To gain insight into these relationships, comprehensive gastrointestinal health studies, integrative analyses of host–microbe–gut interactions, and a deeper understanding of specific microbial taxa and functions’ gut-related functions are necessary.

Lastly, it is important to address the impact of the microbiome on musculoskeletal health and musculoskeletal disorders. The microbiome has been implicated in influencing musculoskeletal physiology, immune responses, and musculoskeletal conditions such as arthritis and musculoskeletal infections [414,415,416,417,418]. To understand these interactions comprehensively, musculoskeletal health studies integrating host–microbe–musculoskeletal interactions are needed.

## 8. Future Directions for Research in Understanding Microbiome and Infectious Disease Pathogenesis

One of the key areas of future research in unraveling the role of the microbiome in infectious disease pathogenesis is the identification of specific microbial species or communities that contribute to disease development. Advanced sequencing technologies, such as metagenomics and metatranscriptomics, can be used in combination with functional studies to understand how these microbes interact with the host immune system and contribute to disease progression. Another important direction for future research is the exploration of the host–microbiome interaction in infectious diseases. Understanding how the host immune system responds to changes in the microbiota during infection is critical for developing effective treatment strategies. We can investigate the role of specific immune cells, such as macrophages and dendritic cells, in modulating the host response to microbial invasion. Additionally, studying the communication between the microbiome and the host immune system through signaling molecules like short-chain fatty acids and metabolites can provide valuable insights into disease pathogenesis. Some future research directions have been tabulated and are shown in Table 3.

Future research should also focus on elucidating the impact of antibiotic use on the microbiome and its role in infectious diseases. Antibiotics can disrupt the natural balance of the microbiota, leading to dysbiosis and increased susceptibility to infections. Investigating how antibiotics alter the composition and function of the microbiome, as well as their long-term effects on disease outcomes, can guide the development of targeted therapies that minimize disruption while effectively treating infections. There is a need for research to explore novel treatment strategies that harness the potential of the microbiome to prevent or treat infectious diseases. Probiotics and FMT have shown promise for certain infections, but their efficacy and safety need further investigation. Developing personalized approaches based on an individual’s unique microbiota composition and immune response could lead to more effective treatment outcomes. Integrating knowledge from other fields such as immunology, genomics, and bioinformatics will be crucial for advancing these research directions.

One important area for future research in treatment strategies for infectious diseases is the development of targeted antimicrobial therapies. Traditional broad-spectrum antibiotics can lead to drug-resistant strains and disrupt the commensal microbiota. By identifying specific microbial species or virulence factors responsible for infection, we can design therapies that selectively target these pathogens while preserving the beneficial components of the microbiome. In addition to antimicrobial therapies, future research should focus on immunomodulatory strategies for infectious diseases. Manipulating the host immune response can enhance pathogen clearance and reduce tissue damage caused by inflammation. Novel approaches such as immunotherapies targeting immune checkpoints or engineering probiotics to produce specific immune-modulating molecules hold great potential for improving treatment outcomes. In addition, exploring the use of bacteriophages as an alternative or adjunctive therapy for infectious diseases is another direction for future research. Bacteriophages offer advantages over antibiotics, including their specificity for target bacteria and lower likelihood of resistance development. However, further research is needed to better understand their safety profile, optimal dosing, and potential interactions with the host–microbiota.

Research efforts should also be directed toward improving diagnostic tools for infectious diseases. Rapid and accurate identification of pathogens is crucial for timely treatment decisions. Advances in technologies such as next-generation sequencing and point-of-care devices hold promise for more sensitive and specific diagnostic methods. Additionally, integrating metagenomic approaches into routine diagnostics can provide a more comprehensive understanding of polymicrobial infections and guide appropriate treatment selection. Future research should focus on identifying key microbial biomarkers associated with resistance or susceptibility to infection. By understanding the specific microbial factors that contribute to disease outcomes, researchers can develop interventions that modulate the microbiome to promote resilience against pathogens.

The use of synthetic biology approaches holds great promise in the field of microbiome research. Researchers can engineer microbes to produce therapeutic molecules or enhance host immune responses. For example, genetically modified probiotics can be designed to secrete AMPs, or anti-inflammatory compounds, providing localized protection against infection.

Personalized medicine approaches aim to tailor treatment strategies based on an individual’s unique characteristics, including their microbiome composition and immune response. Future research should focus on identifying microbial biomarkers that can predict disease susceptibility, treatment response, and potential adverse effects. Integrating metagenomic data with clinical parameters can help develop predictive models to guide personalized treatment decisions.

Advancements in high-throughput sequencing technologies and computational tools offer opportunities to develop microbiome-based diagnostic platforms. Detecting specific microbial signatures or dysbiosis patterns could aid in the early detection and accurate diagnosis of infectious diseases. Additionally, monitoring changes in the microbiota during treatment can provide valuable information on treatment response and inform adjustments in therapeutic strategies.

Understanding how the microbiota community influences vaccine responses is another important area for future research. Exploring the use of microbial adjuvants or immunomodulatory molecules derived from the microbiome can enhance vaccine efficacy. Studies on the gut-lung axis can elucidate the role of the microbiome in respiratory vaccine responses and inform the development of improved vaccines against respiratory pathogens.

Investigating the role of the microbiome in antibiotic resistance and treatment outcomes is crucial. Future research should investigate how specific microbial species or community compositions influence antibiotic resistance gene transfer and the development of multidrug-resistant strains. Additionally, studying the interplay between the microbiome and antibiotic treatment in different infectious diseases can provide insights into optimizing antibiotic regimens and minimizing resistance development.

The impact of the microbiome on infectious diseases extends beyond individual health and has implications for global health. Future research should focus on understanding the influence of environmental factors on the microbiome and its role in infectious diseases. This knowledge can help develop targeted interventions and public health strategies to reduce disease burden in specific populations or regions.

Investigating the role of the microbiome in infectious diseases prevalent in low-resource settings is crucial. Understanding how alterations in the microbiota contribute to disease susceptibility and treatment outcomes in resource-limited settings can guide the development of cost-effective interventions. This research can inform the implementation of microbiome-based interventions in low-income communities where access to traditional healthcare resources may be limited.

Combination therapy holds great potential in the field of infectious diseases. Future research should focus on exploring the synergistic effects of combining microbiome-targeted interventions with traditional antimicrobial therapies. Additionally, developing standardized protocols and guidelines for microbiome-based interventions will ensure safe and effective implementation in healthcare settings.

Future research should focus on investigating the role of the microbiome in infectious diseases from a One Health perspective. By adopting a holistic approach that considers the microbiome across different domains, researchers can develop comprehensive strategies for disease prevention, control, and management.

### 8.1. Future Directions for Research in Microbiome and Novel Therapeutic Targets

Future research should focus on identifying novel therapeutic targets within the microbiome that can be exploited for the development of innovative treatments for infectious diseases. This can involve studying the functional capacities of specific microbial species or communities and their interactions with the host immune system. By understanding the mechanisms by which certain microbes contribute to disease pathogenesis, researchers can identify potential therapeutic targets, such as virulence factors or metabolic pathways, that can be targeted with novel drugs or biologics. Additionally, investigating microbial-host interactions at the molecular level can reveal key signaling pathways or host receptors that can be modulated to influence disease outcomes. The discovery of new therapeutic targets within the microbiome can open up exciting avenues for the development of more precise and effective interventions.

Furthermore, future research should explore the role of the microbiome in the development of host immune memory against infectious diseases. Immune memory is crucial for long-term protection against recurrent infections, and recent evidence suggests that the microbiome plays a significant role in shaping the immune system’s ability to mount effective memory responses. Investigating how the microbiota community influences the development, maintenance, and functionality of immune memory cells, such as memory T cells and B cells, can provide insights into strategies for enhancing vaccine efficacy and long-term protection. Understanding the mechanisms by which the microbiome influences immune memory can guide the development of interventions that modulate the microbiota to promote robust and lasting immune responses against pathogens.

### 8.2. Future Directions for Research in Microbiome and Therapeutic Monitoring

In addition to understanding the role of the microbiome in infectious diseases pathogenesis, future research should focus on developing methods for monitoring and assessing the therapeutic response to microbiome-based interventions. This includes the development of non-invasive biomarkers that can accurately reflect changes in the composition and function of the microbiota during treatment. Researchers can investigate the use of metagenomic, metatranscriptomic, or metabolomic approaches to identify microbial signatures or functional pathways associated with treatment response or resistance. Furthermore, the development of advanced imaging techniques that can visualize and track microbial colonization or clearance within specific host tissues can provide valuable insights into treatment efficacy. Implementing robust and reliable methods for therapeutic monitoring will enable clinicians to assess treatment outcomes, optimize interventions, and make informed decisions regarding patient management.

Moreover, future research should explore the potential of microbiome-based interventions in preventing infectious diseases. While much of the focus has been on treating established infections, there is a growing interest in harnessing the potential of the microbiome for preventive strategies. This can involve the use of probiotics, prebiotics, or postbiotics to modulate the microbiota and enhance host defenses against pathogens. Additionally, understanding how lifestyle factors, such as diet, exercise, or exposure to environmental factors, influence the microbiome and disease susceptibility can inform public health interventions aimed at reducing the risk of infectious diseases. By integrating preventive approaches that target the microbiome into public health strategies, it may be possible to reduce the burden of infectious diseases at the population level.

### 8.3. Future Directions for Research in Microbiome and Microbiota Engineering

The field of microbiota engineering holds promise for developing innovative approaches to modulate the composition and function of the microbiome for therapeutic purposes. Future research should focus on advancing technologies for precise manipulation of the microbiota, such as CRISPR-based methods or targeted delivery systems for microbial therapeutics. Researchers can explore the potential of engineering probiotics or commensal microbes to deliver specific therapeutic payloads, modulate host immune responses, or outcompete pathogenic species. Additionally, investigating the use of synthetic communities of microbes designed to perform specific functions, such as colonization resistance or the production of antimicrobial compounds, can open up new avenues for microbiome-based interventions.

Furthermore, understanding the dynamics of microbial communities in the context of infectious diseases is crucial for developing interventions that promote microbiome resilience and stability. Future research should focus on elucidating the factors that influence microbiome resilience in the face of perturbations caused by infections or antibiotic treatments. This can involve studying the ecological principles that govern microbial community assembly, succession, and stability, as well as identifying keystone species or functional pathways that contribute to microbiome robustness. Developing strategies to promote microbial diversity, functional redundancy, and ecological balance within the microbiota can enhance its ability to resist dysbiosis and prevent pathogen colonization.

### 8.4. Future Directions for Research in Microbiome and Long-Term Outcomes

An important area for future research is the investigation of the long-term effects of microbiome alterations on infectious diseases. While much of the focus has been on immediate disease pathogenesis and treatment, understanding the lasting impact of microbiota perturbations is crucial for informing long-term care strategies. Researchers can explore the role of the microbiome in shaping host immune memory, susceptibility to recurrent infections, and the development of chronic sequelae following acute infections. Longitudinal studies tracking changes in the microbiota composition and function over extended periods can provide insights into the evolving relationship between the microbiome and infectious disease outcomes.

Moreover, future research should focus on elucidating the role of the microbiome in modulating the efficacy and safety of vaccines for infectious diseases. Vaccines are a cornerstone of preventive medicine, and understanding how the microbiota community influences vaccine responses can guide the development of more effective vaccination strategies. Researchers can investigate how alterations in the microbiome impact vaccine immunogenicity, durability of immune responses, and vaccine efficacy across different populations. Additionally, studying the influence of the microbiome on vaccine reactogenicity and adverse events can inform strategies to optimize vaccine safety and tolerability. Integrating microbiome assessments into vaccine trials and post-marketing surveillance efforts can provide valuable insights into optimizing vaccine performance.

### 8.5. Future Directions for Research in Microbiome and Computational Modeling

Advancements in computational modeling offer exciting opportunities for understanding the complex dynamics of the microbiome and its interactions with infectious diseases. Future research should focus on developing predictive models that integrate multi-omics data to simulate the behavior of microbial communities during infection and treatment. These models can provide valuable insights into the mechanisms underlying disease pathogenesis, responses to antimicrobial therapies, and the impact of microbiome-targeted interventions. Additionally, computational approaches can be used to identify key microbial biomarkers or predictive signatures associated with disease progression, treatment response, and long-term outcomes, enabling the development of precision medicine approaches for infectious diseases.

Furthermore, future research should explore the application of machine learning techniques to analyze large-scale microbiome datasets and identify patterns associated with infectious diseases. These approaches can aid in uncovering novel microbial biomarkers, identifying microbial–host interaction networks, and predicting treatment outcomes. Additionally, leveraging machine learning algorithms for personalized risk prediction and treatment optimization based on individual microbiome profiles holds promise for improving clinical decision-making in infectious diseases. Integrating computational modeling and machine learning approaches into microbiome research can enhance our ability to extract meaningful insights from complex biological data and translate them into actionable strategies for disease management.

### 8.6. Future Directions for Research in Microbiome and Therapeutic Modulation

Future research should explore the potential of targeted modulation of the microbiome to restore homeostasis and promote resilience against infectious diseases. This can involve the development of precision interventions that selectively target dysbiotic microbial communities or bolster beneficial microbial populations. Researchers can investigate the use of microbial consortia or engineered probiotics designed to outcompete pathogenic species, restore microbial diversity, and enhance colonization resistance. Additionally, understanding how specific dietary components, such as prebiotics or dietary fibers, influence the microbiome and host–microbiota interactions can inform the development of nutritional interventions aimed at promoting a healthy microbiome and reducing susceptibility to infections. Exploring the potential of precision microbiome modulation approaches can lead to the development of personalized interventions that restore microbial balance and enhance host defenses against pathogens.

Furthermore, future research should focus on investigating the role of the microbiome in shaping systemic immune responses and inflammation during infectious diseases. The gut microbiota community, in particular, plays a crucial role in modulating immune homeostasis and systemic inflammation. Understanding how microbial metabolites, such as short-chain fatty acids and secondary bile acids, influence immune cell function, cytokine production, and tissue inflammation can provide insights into strategies for modulating host immune responses to enhance pathogen clearance and reduce tissue damage. Additionally, exploring the crosstalk between the gut microbiota and systemic immune compartments, such as the lung or skin, can inform the development of interventions that target microbiome–immune interactions to mitigate systemic inflammatory responses during infections. Integrating knowledge from immunology and microbiome research will be crucial for unraveling the complex interplay between the microbiome and systemic immunity in infectious diseases.

Future research in the field of microbiome and host–microbe interactions should focus on elucidating the mechanisms by which the host immune system interacts with the microbiome to shape responses to infectious diseases. This includes understanding how the immune system recognizes and responds to specific microbial components, such as pathogen-associated molecular patterns (PAMPs) or microbial metabolites. Additionally, exploring the influence of the microbiome on adaptive immune cell differentiation, tolerance induction, and memory formation can inform strategies for modulating immune responses to enhance protection against pathogens. Integrating knowledge from immunology and microbiome research will be crucial for unraveling the complex interplay between host immunity and the microbiome in infectious diseases.

In the context of HAIs, future research should focus on understanding how alterations in the hospital microbiota influence infection risk and treatment outcomes. This can involve investigating the use of probiotics, prebiotics, or environmental interventions aimed at promoting a healthy hospital microbiome and reducing the colonization and transmission of multidrug-resistant pathogens. Additionally, studying the impact of infection control measures, such as antimicrobial stewardship programs and environmental cleaning protocols, on the hospital microbiome can provide insights into optimizing strategies for preventing HAIs. Exploring microbiome-based approaches for infection prevention in healthcare settings has the potential to reduce the burden of nosocomial infections and improve patient outcomes.

In the context of chronic infectious diseases, future research should focus on understanding the role of the microbiome in disease persistence and progression. This includes investigating the impact of chronic infections on the composition and function of the microbiota, as well as the potential for the microbiome to influence disease outcomes and treatment responses. Additionally, studying the role of microbial persistence and adaptation within the host–microbiota can inform strategies for targeting persistent infections and preventing disease relapse. Understanding the microbiome’s role in chronic infectious diseases has the potential to guide the development of interventions aimed at modulating microbial communities to promote the resolution of chronic infections and mitigate long-term health consequences.

In pediatric infectious diseases, future research should focus on understanding the development and maturation of the microbiome during early childhood and its influence on susceptibility to infections. This includes investigating how early-life microbial exposures shape the establishment of the infant microbiota and modulate immune development. Additionally, studying the impact of early-life microbiome alterations on the risk of childhood infections and long-term health outcomes can inform interventions aimed at preserving microbial diversity and promoting immune resilience in pediatric populations.

In geriatric infectious diseases, future research should focus on understanding the impact of aging on the microbiome and its influence on susceptibility to infections in older adults. This includes investigating age-related changes in the composition and function of the microbiota, as well as their implications for immune function and resilience against infections. Additionally, studying the impact of microbiome alterations on the risk of geriatric infections, as well as their association with frailty and comorbidities, can inform interventions aimed at preserving microbial diversity and promoting immune resilience in older populations.

Future research should also explore the potential of microbiome-targeted immune-modulating therapies for infectious diseases. This includes investigating the use of microbial-based immunomodulatory agents to regulate immune cell function and enhance host defenses. Additionally, understanding how specific microbial metabolites or signaling molecules influence immune regulatory pathways can inform interventions that modulate host–microbiota interactions to mitigate hyperinflammation and tissue pathology.

Future research in the field of the microbiome and interactions with the virome should focus on several key areas. Firstly, investigating the combined role of the microbiome and virome in infectious diseases is crucial. This involves exploring the impact of viral infections on the composition and function of the microbiota, as well as the potential for interactions between phages, bacteriophages, and eukaryotic viruses to influence disease outcomes. Understanding the crosstalk between the virome and the microbiome in modulating host immune responses and disease susceptibility can provide insights into strategies for targeting viral–microbial interactions to enhance protective immunity against pathogens. Additionally, exploring the combined role of the microbiome and virome has the potential to guide the development of interventions aimed at modulating microbial–viral communities to reduce susceptibility to infections and promote optimal health.

In addition, future research should focus on exploring the potential of leveraging the virome for therapeutic interventions in infectious diseases. This can involve investigating the use of phage therapy, viral immunomodulatory agents, or engineered viral vectors to modulate microbial communities and host immune responses. Precision virome modulation approaches can be explored to mitigate viral infections, restore microbial balance, and promote immune resilience against common pathogens. Understanding how specific viral components or signaling molecules influence microbial–viral interactions and immune function can inform the development of interventions that modulate host–microbiota–virome interactions to promote optimal health outcomes. Exploring virome-based approaches for managing infectious diseases has the potential to provide innovative strategies for addressing infectious diseases and enhancing host defenses against pathogens.

Exploring the role of microbial metabolites in infectious diseases and their potential as therapeutic targets is also an interesting area of research. Microbial metabolites, such as short-chain fatty acids, secondary bile acids, and various signaling molecules, play a crucial role in modulating host–microbiota interactions and immune responses. Investigating the impact of microbial metabolites on immune cell function, inflammation, and tissue homeostasis during infections is important. Understanding how specific microbial metabolites influence disease progression and treatment responses can provide insights into strategies for modulating host immune responses and promoting pathogen clearance. Additionally, exploring the potential of targeting microbial metabolites to modulate the microbiome and enhance protective immunity against pathogens has the potential to inform the development of novel interventions for infectious diseases. Furthermore, investigating the influence of microbial metabolite profiles on disease outcomes and treatment efficacy can provide valuable insights into the development of diagnostic and therapeutic strategies. Integrating microbial metabolite assessments into clinical practice can provide valuable information for guiding treatment decisions and monitoring therapeutic interventions. Exploring the potential of microbial metabolites as biomarkers has the potential to revolutionize diagnostic and treatment approaches for infectious diseases.

Future research should focus on integrating multi-omics data, including genomics, transcriptomics, proteomics, and metabolomics, to comprehensively understand the role of the microbiome in infectious diseases. Combining data from multiple omics layers can provide a holistic understanding of the molecular interactions between the host, microbiome, and pathogens during infections. This integrative approach can provide insights into the functional activities of microbial communities, host immune responses, and disease pathways. Additionally, integrating multi-omics data can facilitate the identification of novel biomarkers, therapeutic targets, and predictive signatures associated with disease progression and treatment responses. Exploring multi-omics integration has the potential to revolutionize our understanding of infectious diseases and inform the development of precision medicine approaches.

Moreover, longitudinal studies tracking the dynamics of the microbiome in individuals over time can provide valuable insights into temporal changes in microbial composition and their association with infectious diseases. Long-term cohort studies can offer a deeper understanding of how the microbiome responds to infectious challenges, the stability of microbial communities post-infection, and potential long-term impacts on host health. Integrating longitudinal microbiome data with clinical parameters can facilitate the identification of microbial signatures associated with disease progression, treatment response, and long-term health outcomes. Furthermore, large-scale population-based studies can elucidate the impact of environmental and sociodemographic factors on the microbiome and infectious diseases.

Research should also focus on translating insights from microbiome research into clinical applications by developing targeted interventions aimed at modulating the microbiome to prevent and treat infectious diseases. Investigating microbiome modulation through probiotics, prebiotics, postbiotics, and FMT as therapeutic strategies can provide valuable insights into the development of microbiome-based interventions. Additionally, exploring precision approaches for targeting dysbiotic microbial communities and enhancing colonization resistance against pathogens can inform the design of novel therapeutics. Investigating combination therapies involving microbiome-based interventions with traditional antimicrobial therapies can improve treatment outcomes for infectious diseases.

Ethical considerations related to microbiome-based interventions should also be addressed in future research. Examining the ethical implications of manipulating the microbiome for therapeutic purposes is essential in terms of consent, privacy, and equity. Understanding the potential risks and benefits of microbiome-based interventions can inform ethical guidelines and regulatory frameworks. Community engagement and public education initiatives can raise awareness about the microbiome’s role in infectious diseases. Effective communication strategies are needed to ensure informed decision-making and promote equitable access to microbiome-based interventions.

Global collaboration and data sharing are crucial for advancing our understanding of the microbiome’s role in infectious diseases. International partnerships and initiatives can harmonize microbiome research and facilitate cross-disciplinary collaboration. Interdisciplinary training programs can cultivate a new generation of researchers with a comprehensive understanding of microbiome–host interactions. Long-term health outcomes associated with microbiome alterations due to infectious diseases should also be investigated to develop strategies that mitigate any long-term health consequences. Microbiome-based interventions have potential for promoting long-term health and resilience against recurrent infections.

## 9. Conclusions

In conclusion, this review article has highlighted the significant role of the microbiome in the pathogenesis of infectious diseases and its potential as a target for innovative treatment strategies. The findings discussed throughout the article have shed light on the intricate relationship between the microbiome and infectious disease progression. Key findings indicate that alterations in the microbiome composition can influence the susceptibility, severity, and outcome of infectious diseases. The microbiome can modulate host immune responses, impact the efficacy of antimicrobial therapies, and even contribute to the development of drug resistance. Understanding these interactions is crucial for devising effective treatment approaches. Moreover, considering the microbiome in infectious disease research has the potential to revolutionize our understanding of pathogenesis and treatment strategies. By targeting the microbiome, we may be able to develop novel therapies that can restore microbial balance, enhance host immune responses, and improve patient outcomes. In light of these findings, it is evident that the microbiome plays a pivotal role in infectious disease pathogenesis and treatment. It is imperative for researchers, clinicians, and policymakers to recognize the importance of considering the microbiome in their work. By doing so, we can unlock new avenues for combating infectious diseases and ultimately improve global health outcomes.

## Figures and Tables

**Table 2:** Challenges and subtypes facing the microbiome in infectious diseases.

Challenge	Description and Subtypes
1. Complexity of the Microbiome	- Vast community of microorganisms - Interactions between microorganisms and host - Dynamic nature and variability in the microbiome
2. Analytical Tools and Techniques	- Limitations of culture-based methods - Challenges of high-throughput sequencing technologies - Ethical considerations in sample access and analysis
3. Understanding Various Microorganisms	- Limited knowledge of interactions with host immune system - Difficulties in distinguishing pathogenic and commensal microorganisms - Logistical challenges of longitudinal studies
4. Translation into Clinical Applications	- Individual variations and evolving regulatory frameworks - Establishing causality and developing effective interventions - Standardization of methodologies and protocols
5. Specialized Techniques and Interdisciplinary Collaborations	- Need for specialized techniques like metatranscriptomics and metabolomics - Challenges in building effective interdisciplinary collaborations - Heterogeneity within infectious diseases and disease progression
6. Ethical, Environmental, and Public Health Considerations	- Influence of environmental factors on microbial communities and disease outcomes - Impact of antibiotic resistance and usage on the microbiome - Addressing disparities in microbiome research and ensuring inclusivity across diverse populations
7. Data Integration, Interpretation, and Standardization	- Challenges in data storage, management, and analysis - Variations in study design, sampling protocols, and bioinformatics pipelines - Robust computational infrastructure and bioinformatics expertise
8. Host–Microbiome Interactions and Immune Responses and Impact on Reproductive, Respiratory, and Metabolic Health, Oral, Gastrointestinal, and Dermatological Challenges, and Musculoskeletal and Public Health Considerations	- Influence of host genetics, immune status, and underlying health conditions - Impact of the microbiome on vaccine responses and efficacy - Role of the microbiome in modulating immune responses during infectious diseases. - Influence of microbiome on different systems such as reproductive, respiratory, metabolic, oral, gastrointestinal, skin, and musculoskeletal health and disorders
9. Regulatory and Policy Challenges	-Developing evidence-based policies, regulatory guidelines, and ethical frameworks - Ensuring effective communication and knowledge dissemination across diverse stakeholders - Legal and ethical considerations related to microbiome-based interventions

**Table 3:** Future research directions in understanding the microbiome and infectious diseases.

Research Area	Key Focus
Role of Microbiome in Infectious Disease Pathogenesis	- Identification of specific microbial species or communities contributing to disease development - Host–microbiome interactions in infectious diseases - Impact of antibiotic use on the microbiome in infectious diseases - Novel treatment strategies utilizing the potential of the microbiome - Targeted antimicrobial and immunomodulatory therapies - Improvement in diagnostic tools for infectious diseases - Synthetic biology approaches for microbiome research
Microbiome and Novel Therapeutic Targets	- Identifying functional capacities of specific microbial species or communities - Understanding the role of the microbiome in the development of host immune memory against infectious diseases
Microbiome and Therapeutic Monitoring	- Developing methods for monitoring and assessing the therapeutic response to microbiome-based interventions - Exploring potential of microbiome-based interventions in preventing infectious diseases
Microbiome and Microbiota Engineering	- Advancing technologies for precise manipulation of the microbiota - Understanding dynamics of microbial communities in the context of infectious diseases
Microbiome and Long-term Outcomes	- Investigating the long-term effects of microbiome alterations on infectious diseases - Elucidating the role of the microbiome in modulating the efficacy and safety of vaccines for infectious diseases
Microbiome and Computational Modeling	- Developing predictive models that integrate multi-omic data to simulate the behavior of microbial communities during infection and treatment - Application of machine learning techniques to analyze large-scale microbiome datasets
Microbiome and Therapeutic Modulation	- Targeted modulation of the microbiome to restore homeostasis and promote resilience against infectious diseases - Investigating the role of the microbiome in shaping systemic immune responses and inflammation during infectious diseases
Microbiome and Host–Microbe Interactions	- Understanding how the host immune system interacts with the microbiome to shape responses to infectious diseases - Impact of alterations in the hospital microbiota on infection risk and treatment outcomes
Microbiome in Specific Populations	- Understanding the development and maturation of the microbiome during early childhood and its influence on susceptibility to infections in pediatric populations - Impact of aging on the microbiome and its influence on susceptibility to infections in older adults

## Data Availability

All data are included in the review article paper.

## References

[1] El-Sayed A., Aleya L., Kamel M. (2021). Microbiota and epigenetics: Promising therapeutic approaches?. Environ. Sci. Pollut. Res. Int..

[2] El-Sayed A., Aleya L., Kamel M. (2021). Microbiota’s role in health and diseases. Environ. Sci. Pollut. Res..

[3] El-Sayed A., Aleya L., Kamel M. (2021). The link among microbiota, epigenetics, and disease development. Environ. Sci. Pollut. Res..

[4] Costantini C., Nunzi E., Spolzino A., Merli F., Facchini L., Spadea A., Melillo L., Codeluppi K., Marchesi F., Marchesini G. (2022). A High-Risk Profile for Invasive Fungal Infections Is Associated with Altered Nasal Microbiota and Niche Determinants. Infect. Immun..

[5] Hurst J.H., Heston S.M., Kelly M.S. (2023). Host microbiome-pathogen interactions in pediatric infections. Curr. Opin. Infect. Dis..

[6] Levy M., Thaiss C.A., Zeevi D., Dohnalová L., Zilberman-Schapira G., Mahdi J.A., David E., Savidor A., Korem T., Herzig Y. (2015). Microbiota-Modulated Metabolites Shape the Intestinal Microenvironment by Regulating NLRP6 Inflammasome Signaling. Cell.

[7] Sun J., Ince M.N., Abraham C., Barrett T., Brenner L.A., Cong Y., Dashti R., Dudeja P.K., Elliott D., Griffith T.S. (2023). Modulating microbiome-immune axis in the deployment-related chronic diseases of Veterans: Report of an expert meeting. Gut Microbes.

[8] Sun Y., Zhang S., Nie Q., He H., Tan H., Geng F., Ji H., Hu J., Nie S. (2023). Gut firmicutes: Relationship with dietary fiber and role in host homeostasis. Crit. Rev. Food Sci. Nutr..

[9] Rajilić-Stojanović M., de Vos W.M. (2014). The first 1000 cultured species of the human gastrointestinal microbiota. FEMS Microbiol. Rev..

[10] Dewhirst F.E., Chen T., Izard J., Paster B.J., Tanner A.C.R., Yu W.-H., Lakshmanan A., Wade W.G. (2010). The human oral microbiome. J. Bacteriol..

[11] Luqman A., Zabel S., Rahmdel S., Merz B., Gruenheit N., Harter J., Nieselt K., Götz F. (2020). The Neuromodulator-Encoding sadA Gene Is Widely Distributed in the Human Skin Microbiome. Front. Microbiol..

[12] Ceccarani C., Foschi C., Parolin C., D’Antuono A., Gaspari V., Consolandi C., Laghi L., Camboni T., Vitali B., Severgnini M. (2019). Diversity of vaginal microbiome and metabolome during genital infections. Sci. Rep..

[13] Ravel J., Gajer P., Abdo Z., Schneider G.M., Koenig S.S.K., McCulle S.L., Karlebach S., Gorle R., Russell J., Tacket C.O. (2011). Vaginal microbiome of reproductive-age women. Proc. Natl. Acad. Sci. USA.

[14] Diao W., Shen N., Du Y., Qian K., He B. (2017). Characterization of throat microbial flora in smokers with or without COPD. Int. J. Chron. Obstruct. Pulmon. Dis..

[15] Tang Z., Yang S., He Z. (2022). Defining the baseline of pulmonary microbiota in healthy populations and influencing factors. Highlights Sci. Eng. Technol..

[16] Hooper L.V., Littman D.R., Macpherson A.J. (2012). Interactions between the microbiota and the immune system. Science.

[17] Altveş S., Yildiz H.K., Vural H.C. (2020). Interaction of the microbiota with the human body in health and diseases. Biosci. Microbiota Food Health.

[18] Alshehri D., Saadah O., Mosli M., Edris S., Alhindi R., Bahieldin A. (2021). Dysbiosis of gut microbiota in inflammatory bowel disease: Current therapies and potential for microbiota-modulating therapeutic approaches. Bosn. J. Basic Med. Sci..

[19] Carding S., Verbeke K., Vipond D.T., Corfe B.M., Owen L.J. (2015). Dysbiosis of the gut microbiota in disease. Microb. Ecol. Health Dis..

[20] Schlechte J., Zucoloto A.Z., Yu I.-L., Doig C.J., Dunbar M.J., McCoy K.D., McDonald B. (2023). Dysbiosis of a microbiota-immune metasystem in critical illness is associated with nosocomial infections. Nat. Med..

[21] Seekatz A.M., Safdar N., Khanna S. (2022). The role of the gut microbiome in colonization resistance and recurrent Clostridioides difficile infection. Ther. Adv. Gastroenterol..

[22] Schubert A.M., Sinani H., Schloss P.D. (2015). Antibiotic-Induced Alterations of the Murine Gut Microbiota and Subsequent Effects on Colonization Resistance against Clostridium difficile. mBio.

[23] Moffatt M.F., Cookson W.O. (2017). The lung microbiome in health and disease. Clin. Med..

[24] Dumas A., Bernard L., Poquet Y., Lugo-Villarino G., Neyrolles O. (2018). The role of the lung microbiota and the gut-lung axis in respiratory infectious diseases. Cell. Microbiol..

[25] Wiesenfeld H.C., Hillier S.L., Krohn M.A., Landers D.V., Sweet R.L. (2003). Bacterial vaginosis is a strong predictor of Neisseria gonorrhoeae and Chlamydia trachomatis infection. Clin. Infect. Dis..

[26] Ahrens P., Andersen L.O., Lilje B., Johannesen T.B., Dahl E.G., Baig S., Jensen J.S., Falk L. (2020). Changes in the vaginal microbiota following antibiotic treatment for *Mycoplasma genitalium*, *Chlamydia trachomatis* and bacterial vaginosis. PLoS ONE.

[27] Ong I.J., Loo K.-Y., Law L.N.-S., Law J.W.-F., Tan L.T.-H., Letchumanan V. (2023). Exploring the impact of Helicobacter pylori and potential gut microbiome modulation. Prog. Microbes Mol. Biol..

[28] Gomez-Ramirez U., Valencia-Mayoral P., Mendoza-Elizalde S., Murillo-Eliosa J.R., Solórzano Santos F., Contreras-Rodríguez A., Zúñiga G., Aguilar-Rodea P., Jiménez-Rojas V.L., Vigueras Galindo J.C. (2021). Role of Helicobacter pylori and Other Environmental Factors in the Development of Gastric Dysbiosis. Pathogens.

[29] Zhang L., Zhao M., Fu X. (2023). Gastric microbiota dysbiosis and *Helicobacter pylori* infection. Front. Microbiol..

[30] Noto J.M., Peek R.M. (2017). The gastric microbiome, its interaction with *Helicobacter pylori*, and its potential role in the progression to stomach cancer. PLoS Pathog..

[31] Dash N.R., Khoder G., Nada A.M., Al Bataineh M.T. (2019). Exploring the impact of *Helicobacter pylori* on gut microbiome composition. PLoS ONE.

[32] Gallagher K., Catesson A., Griffin J.L., Holmes E., Williams H.R.T. (2021). Metabolomic Analysis in Inflammatory Bowel Disease: A Systematic Review. J. Crohn’s Colitis.

[33] Fitz-Gibbon S., Tomida S., Chiu B.-H., Nguyen L., Du C., Liu M., Elashoff D., Erfe M.C., Loncaric A., Kim J. (2013). Propionibacterium acnes strain populations in the human skin microbiome associated with acne. J. Investig. Dermatol..

[34] Teng V.C., Esti P.K. (2021). Skin microbiome dysbiosis in leprosy cases. Int. J. Res. Dermatol..

[35] Mekadim C., Skalnikova H.K., Cizkova J., Cizkova V., Palanova A., Horak V., Mrazek J. (2022). Dysbiosis of skin microbiome and gut microbiome in melanoma progression. BMC Microbiol..

[36] Jani A.J., Briggs C.J. (2014). The pathogen *Batrachochytrium dendrobatidis* disturbs the frog skin microbiome during a natural epidemic and experimental infection. Proc. Natl. Acad. Sci. USA.

[37] Lamont R.J., Koo H., Hajishengallis G. (2018). The oral microbiota: Dynamic communities and host interactions. Nat. Rev. Microbiol..

[38] Lev-Sagie A., de Seta F., Verstraelen H., Ventolini G., Lonnee-Hoffmann R., Vieira-Baptista P. (2022). The Vaginal Microbiome: II. Vaginal Dysbiotic Conditions. J. Low. Genit. Tract. Dis..

[39] Holdcroft A.M., Ireland D.J., Payne M.S. (2023). The Vaginal Microbiome in Health and Disease-What Role Do Common Intimate Hygiene Practices Play?. Microorganisms.

[40] Kindinger L.M., MacIntyre D.A., Lee Y.S., Marchesi J.R., Smith A., McDonald J.A.K., Terzidou V., Cook J.R., Lees C., Israfil-Bayli F. (2016). Relationship between vaginal microbial dysbiosis, inflammation, and pregnancy outcomes in cervical cerclage. Sci. Transl. Med..

[41] Gordon J.I. (2012). Honor thy gut symbionts redux. Science.

[42] Dizzell S., Nazli A., Reid G., Kaushic C. (2019). Protective Effect of Probiotic Bacteria and Estrogen in Preventing HIV-1-Mediated Impairment of Epithelial Barrier Integrity in Female Genital Tract. Cells.

[43] Hou L., Yang Y., Sun B., Jing Y., Deng W. (2021). Dietary Fiber, Gut Microbiota, Short-Chain Fatty Acids, and Host Metabolism. Am. J. Life Sci..

[44] Basen M., Kurrer S.E. (2021). A close look at pentose metabolism of gut bacteria. FEBS J..

[45] Caballero S., Pamer E.G. (2015). Microbiota-mediated inflammation and antimicrobial defense in the intestine. Annu. Rev. Immunol..

[46] Zhang N., He Q.-S. (2015). Commensal Microbiome Promotes Resistance to Local and Systemic Infections. Chin. Med. J..

[47] Ivanov I.I., Atarashi K., Manel N., Brodie E.L., Shima T., Karaoz U., Wei D., Goldfarb K.C., Santee C.A., Lynch S.V. (2009). Induction of intestinal Th17 cells by segmented filamentous bacteria. Cell.

[48] Wang Y., Yin Y., Chen X., Zhao Y., Wu Y., Li Y., Wang X., Chen H., Xiang C. (2019). Induction of Intestinal Th17 Cells by Flagellins from Segmented Filamentous Bacteria. Front. Immunol..

[49] Schnupf P., Gaboriau-Routhiau V., Sansonetti P.J., Cerf-Bensussan N. (2017). Segmented filamentous bacteria, Th17 inducers and helpers in a hostile world. Curr. Opin. Microbiol..

[50] Hooper L.V., Macpherson A.J. (2010). Immune adaptations that maintain homeostasis with the intestinal microbiota. Nat. Rev. Immunol..

[51] Frosali S., Pagliari D., Gambassi G., Landolfi R., Pandolfi F., Cianci R. (2015). How the Intricate Interaction among Toll-Like Receptors, Microbiota, and Intestinal Immunity Can Influence Gastrointestinal Pathology. J. Immunol. Res..

[52] Donia M.S., Fischbach M.A. (2015). Small molecules from the human microbiota. Science.

[53] Garcia-Gutierrez E., Mayer M.J., Cotter P.D., Narbad A. (2019). Gut microbiota as a source of novel antimicrobials. Gut Microbes.

[54] Darbandi A., Asadi A., Mahdizade Ari M., Ohadi E., Talebi M., Halaj Zadeh M., Darb Emamie A., Ghanavati R., Kakanj M. (2022). Bacteriocins: Properties and potential use as antimicrobials. J. Clin. Lab. Anal..

[55] Vincent C., Manges A.R. (2015). Antimicrobial Use, Human Gut Microbiota and *Clostridium difficile* Colonization and Infection. Antibiotics.

[56] Sorbara M.T., Pamer E.G. (2019). Interbacterial mechanisms of colonization resistance and the strategies pathogens use to overcome them. Mucosal Immunol..

[57] Guilloteau P., Martin L., Eeckhaut V., Ducatelle R., Zabielski R., van Immerseel F. (2010). From the gut to the peripheral tissues: The multiple effects of butyrate. Nutr. Res. Rev..

[58] Ashida H., Ogawa M., Kim M., Mimuro H., Sasakawa C. (2011). Bacteria and host interactions in the gut epithelial barrier. Nat. Chem. Biol..

[59] Kitamoto S., Nagao-Kitamoto H., Kuffa P., Kamada N. (2016). Regulation of virulence: The rise and fall of gastrointestinal pathogens. J. Gastroenterol..

[60] Lawley T.D., Walker A.W. (2013). Intestinal colonization resistance. Immunology.

[61] Whiteley M., Diggle S.P., Greenberg E.P. (2017). Progress in and promise of bacterial quorum sensing research. Nature.

[62] Ma J., Piao X., Mahfuz S., Long S., Wang J. (2022). The interaction among gut microbes, the intestinal barrier and short chain fatty acids. Anim. Nutr..

[63] Gasaly N., de Vos P., Hermoso M.A. (2021). Impact of Bacterial Metabolites on Gut Barrier Function and Host Immunity: A Focus on Bacterial Metabolism and Its Relevance for Intestinal Inflammation. Front. Immunol..

[64] Parada Venegas D., De La Fuente M.K., Landskron G., González M.J., Quera R., Dijkstra G., Harmsen H.J.M., Faber K.N., Hermoso M.A. (2019). Short Chain Fatty Acids (SCFAs)-Mediated Gut Epithelial and Immune Regulation and Its Relevance for Inflammatory Bowel Diseases. Front. Immunol..

[65] Yoo J.Y., Groer M., Dutra S.V.O., Sarkar A., McSkimming D.I. (2020). Gut Microbiota and Immune System Interactions. Microorganisms.

[66] Stecher B. (2015). The Roles of Inflammation, Nutrient Availability and the Commensal Microbiota in Enteric Pathogen Infection. Metabolism and Bacterial Pathogenesis.

[67] Gerner R.R., Nuccio S.-P., Raffatellu M. (2020). Iron at the host-microbe interface. Mol. Asp. Med..

[68] Lopez C.A., Skaar E.P. (2018). The Impact of Dietary Transition Metals on Host-Bacterial Interactions. Cell Host Microbe.

[69] Santos-Júnior C.D., Der Torossian Torres M., Duan Y., Del Río Á.R., Schmidt T.S.B., Chong H., Fullam A., Kuhn M., Zhu C., Houseman A. (2023). Computational exploration of the global microbiome for antibiotic discovery. bioRxiv.

[70] Brito I.L. (2021). Examining horizontal gene transfer in microbial communities. Nat. Rev. Microbiol..

[71] von Wintersdorff C.J.H., Penders J., van Niekerk J.M., Mills N.D., Majumder S., van Alphen L.B., Savelkoul P.H.M., Wolffs P.F.G. (2016). Dissemination of Antimicrobial Resistance in Microbial Ecosystems through Horizontal Gene Transfer. Front. Microbiol..

[72] Willmann M., Vehreschild M.J.G.T., Biehl L.M., Vogel W., Dörfel D., Hamprecht A., Seifert H., Autenrieth I.B., Peter S. (2019). Distinct impact of antibiotics on the gut microbiome and resistome: A longitudinal multicenter cohort study. BMC Biol..

[73] Luchen C.C., Chibuye M., Spijker R., Simuyandi M., Chisenga C., Bosomprah S., Chilengi R., Schultsz C., Mende D.R., Harris V.C. (2023). Impact of antibiotics on gut microbiome composition and resistome in the first years of life in low- to middle-income countries: A systematic review. PLoS Med..

[74] Hitchings R., Kelly L. (2019). Predicting and Understanding the Human Microbiome’s Impact on Pharmacology. Trends Pharmacol. Sci..

[75] Zhang J., Zhang J., Wang R. (2018). Gut microbiota modulates drug pharmacokinetics. Drug Metab. Rev..

[76] Wilson I.D., Nicholson J.K. (2017). Gut microbiome interactions with drug metabolism, efficacy, and toxicity. Transl. Res..

[77] Enright E.F., Gahan C.G.M., Joyce S.A., Griffin B.T. (2016). The Impact of the Gut Microbiota on Drug Metabolism and Clinical Outcome. Yale J. Biol. Med..

[78] Wan Y., Zuo T. (2022). Interplays between drugs and the gut microbiome. Gastroenterol. Rep..

[79] Desselberger U. (2021). Significance of the Gut Microbiome for Viral Diarrheal and Extra-Intestinal Diseases. Viruses.

[80] Wiertsema S.P., van Bergenhenegouwen J., Garssen J., Knippels L.M.J. (2021). The Interplay between the Gut Microbiome and the Immune System in the Context of Infectious Diseases throughout Life and the Role of Nutrition in Optimizing Treatment Strategies. Nutrients.

[81] Choden T., Cohen N.A. (2022). The gut microbiome and the immune system. Explor. Med..

[82] D’Aquila P., Carelli L.L., de Rango F., Passarino G., Bellizzi D. (2020). Gut Microbiota as Important Mediator Between Diet and DNA Methylation and Histone Modifications in the Host. Nutrients.

[83] Bhat M.I., Kapila R. (2017). Dietary metabolites derived from gut microbiota: Critical modulators of epigenetic changes in mammals. Nutr. Rev..

[84] Miro-Blanch J., Yanes O. (2019). Epigenetic Regulation at the Interplay Between Gut Microbiota and Host Metabolism. Front. Genet..

[85] Ye J., Wu W., Li Y., Li L. (2017). Influences of the Gut Microbiota on DNA Methylation and Histone Modification. Dig. Dis. Sci..

[86] El-Sayed A., Aleya L., Kamel M. (2023). Epigenetics and the role of nutraceuticals in health and disease. Environ. Sci. Pollut. Res..

[87] Sommer M.O.A., Church G.M., Dantas G. (2010). The human microbiome harbors a diverse reservoir of antibiotic resistance genes. Virulence.

[88] Garcia V.R. (2021). Impact of Intrapartum Antibiotic Prophylaxis for Group B Streptococcus on the Term Infant Gut Microbiome: A State of the Science Review. J. Midwifery Women’s Health.

[89] Panwar R.B., Sequeira R.P., Clarke T.B. (2021). Microbiota-mediated protection against antibiotic-resistant pathogens. Genes. Immun..

[90] Zheng D., Liwinski T., Elinav E. (2020). Interaction between microbiota and immunity in health and disease. Cell Res..

[91] Barzegari A., Kheyrolahzadeh K., Hosseiniyan Khatibi S.M., Sharifi S., Memar M.Y., Zununi Vahed S. (2020). The Battle of Probiotics and Their Derivatives Against Biofilms. Infect. Drug Resist..

[92] Miller A.L., Bessho S., Grando K., Tükel Ç. (2021). Microbiome or Infections: Amyloid-Containing Biofilms as a Trigger for Complex Human Diseases. Front. Immunol..

[93] Jamieson A.M. (2015). Influence of the microbiome on response to vaccination. Hum. Vaccin. Immunother..

[94] Daddi L., Dorsett Y., Geng T., Bokoliya S., Yuan H., Wang P., Xu W., Zhou Y. (2023). Baseline Gut Microbiome Signatures Correlate with Immunogenicity of SARS-CoV-2 mRNA Vaccines. Int. J. Mol. Sci..

[95] Amato K.R., Arrieta M.-C., Azad M.B., Bailey M.T., Broussard J.L., Bruggeling C.E., Claud E.C., Costello E.K., Davenport E.R., Dutilh B.E. (2021). The human gut microbiome and health inequities. Proc. Natl. Acad. Sci. USA.

[96] Johnson K.V.-A. (2020). Gut microbiome composition and diversity are related to human personality traits. Hum. Microb. J..

[97] Blum W.E.H., Zechmeister-Boltenstern S., Keiblinger K.M. (2019). Does Soil Contribute to the Human Gut Microbiome?. Microorganisms.

[98] Xu Z., Knight R. (2015). Dietary effects on human gut microbiome diversity. Br. J. Nutr..

[99] Gupta V.K., Paul S., Dutta C. (2017). Geography, Ethnicity or Subsistence-Specific Variations in Human Microbiome Composition and Diversity. Front. Microbiol..

[100] Scepanovic P., Hodel F., Mondot S., Partula V., Byrd A., Hammer C., Alanio C., Bergstedt J., Patin E., Touvier M. (2019). A comprehensive assessment of demographic, environmental, and host genetic associations with gut microbiome diversity in healthy individuals. Microbiome.

[101] (2012). Structure, function and diversity of the healthy human microbiome. Nature.

[102] Zhou Y., Gao H., Mihindukulasuriya K.A., La Rosa P.S., Wylie K.M., Vishnivetskaya T., Podar M., Warner B., Tarr P.I., Nelson D.E. (2013). Biogeography of the ecosystems of the healthy human body. Genome Biol..

[103] Hall M.W., Singh N., Ng K.F., Lam D.K., Goldberg M.B., Tenenbaum H.C., Neufeld J.D., Beiko R.G., Senadheera D.B. (2017). Inter-personal diversity and temporal dynamics of dental, tongue, and salivary microbiota in the healthy oral cavity. npj Biofilms Microbiomes.

[104] Asangba A.E., Mugisha L., Rukundo J., Lewis R.J., Halajian A., Cortés-Ortiz L., Junge R.E., Irwin M.T., Karlson J., Perkin A. (2022). Large Comparative Analyses of Primate Body Site Microbiomes Indicate that the Oral Microbiome Is Unique among All Body Sites and Conserved among Nonhuman Primates. Microbiol. Spectr..

[105] Pamer E.G. (2007). Immune responses to commensal and environmental microbes. Nat. Immunol..

[106] Ciabattini A., Olivieri R., Lazzeri E., Medaglini D. (2019). Role of the Microbiota in the Modulation of Vaccine Immune Responses. Front. Microbiol..

[107] Vlasova A.N., Takanashi S., Miyazaki A., Rajashekara G., Saif L.J. (2019). How the gut microbiome regulates host immune responses to viral vaccines. Curr. Opin. Virol..

[108] Lynn D.J., Benson S.C., Lynn M.A., Pulendran B. (2022). Modulation of immune responses to vaccination by the microbiota: Implications and potential mechanisms. Nat. Rev. Immunol..

[109] Hagan T., Cortese M., Rouphael N., Boudreau C., Linde C., Maddur M.S., Das J., Wang H., Guthmiller J., Zheng N.-Y. (2019). Antibiotics-driven gut microbiome perturbation alters immunity to vaccines in humans. Cell.

[110] Ramakrishna B.S. (2013). Role of the gut microbiota in human nutrition and metabolism. J. Gastroenterol. Hepatol..

[111] Ximenez C., Torres J. (2017). Development of Microbiota in Infants and its Role in Maturation of Gut Mucosa and Immune System. Arch. Med. Res..

[112] Dogra S.K., Kwong Chung C., Wang D., Sakwinska O., Colombo Mottaz S., Sprenger N. (2021). Nurturing the Early Life Gut Microbiome and Immune Maturation for Long Term Health. Microorganisms.

[113] Ficara M., Pietrella E., Spada C., Della Casa Muttini E., Lucaccioni L., Iughetti L., Berardi A. (2020). Changes of intestinal microbiota in early life. J. Matern. Fetal Neonatal Med..

[114] Parkin K., Christophersen C.T., Verhasselt V., Cooper M.N., Martino D. (2021). Risk Factors for Gut Dysbiosis in Early Life. Microorganisms.

[115] Cristofori F., Dargenio V.N., Dargenio C., Miniello V.L., Barone M., Francavilla R. (2021). Anti-Inflammatory and Immunomodulatory Effects of Probiotics in Gut Inflammation: A Door to the Body. Front. Immunol..

[116] Ivanov I.I., Littman D.R. (2011). Modulation of immune homeostasis by commensal bacteria. Curr. Opin. Microbiol..

[117] Postler T.S., Ghosh S. (2017). Understanding the Holobiont: How Microbial Metabolites Affect Human Health and Shape the Immune System. Cell Metab..

[118] Gaudino S.J., Kumar P. (2019). Cross-Talk Between Antigen Presenting Cells and T Cells Impacts Intestinal Homeostasis, Bacterial Infections, and Tumorigenesis. Front. Immunol..

[119] Rooks M.G., Garrett W.S. (2016). Gut microbiota, metabolites and host immunity. Nat. Rev. Immunol..

[120] Salvo-Romero E., Stokes P., Gareau M.G. (2020). Microbiota-immune interactions: From gut to brain. LymphoSign J..

[121] Fung T.C., Olson C.A., Hsiao E.Y. (2017). Interactions between the microbiota, immune and nervous systems in health and disease. Nat. Neurosci..

[122] Foster J.A., Rinaman L., Cryan J.F. (2017). Stress & the gut-brain axis: Regulation by the microbiome. Neurobiol. Stress.

[123] Rutsch A., Kantsjö J.B., Ronchi F. (2020). The Gut-Brain Axis: How Microbiota and Host Inflammasome Influence Brain Physiology and Pathology. Front. Immunol..

[124] Wiley N.C., Dinan T.G., Ross R.P., Stanton C., Clarke G., Cryan J.F. (2017). The microbiota-gut-brain axis as a key regulator of neural function and the stress response: Implications for human and animal health. J. Anim. Sci..

[125] Fock E., Parnova R. (2023). Mechanisms of Blood-Brain Barrier Protection by Microbiota-Derived Short-Chain Fatty Acids. Cells.

[126] Tang W., Zhu H., Feng Y., Guo R., Wan D. (2020). The Impact of Gut Microbiota Disorders on the Blood-Brain Barrier. Infect. Drug Resist..

[127] Mitra S., Dash R., Nishan A.A., Habiba S.U., Moon I.S. (2023). Brain modulation by the gut microbiota: From disease to therapy. J. Adv. Res..

[128] Vernocchi P., Del Chierico F., Putignani L. (2020). Gut Microbiota Metabolism and Interaction with Food Components. Int. J. Mol. Sci..

[129] Rowland I., Gibson G., Heinken A., Scott K., Swann J., Thiele I., Tuohy K. (2018). Gut microbiota functions: Metabolism of nutrients and other food components. Eur. J. Nutr..

[130] Khachatryan Z.A., Ktsoyan Z.A., Manukyan G.P., Kelly D., Ghazaryan K.A., Aminov R.I. (2008). Predominant role of host genetics in controlling the composition of gut microbiota. PLoS ONE.

[131] Bubier J.A., Chesler E.J., Weinstock G.M. (2021). Host genetic control of gut microbiome composition. Mamm. Genome.

[132] Forgie A.J., Fouhse J.M., Willing B.P. (2019). Diet-Microbe-Host Interactions That Affect Gut Mucosal Integrity and Infection Resistance. Front. Immunol..

[133] Hooper L.V., Gordon J.I. (2001). Commensal host-bacterial relationships in the gut. Science.

[134] García-Bayona L., Comstock L.E. (2018). Bacterial antagonism in host-associated microbial communities. Science.

[135] Botía-Sánchez M., Alarcón-Riquelme M.E., Galicia G. (2021). B Cells and Microbiota in Autoimmunity. Int. J. Mol. Sci..

[136] Mishima Y., Oka A., Liu B., Herzog J.W., Eun C.S., Fan T.-J., Bulik-Sullivan E., Carroll I.M., Hansen J.J., Chen L. (2019). Microbiota maintain colonic homeostasis by activating TLR2/MyD88/PI3K signaling in IL-10-producing regulatory B cells. J. Clin. Investig..

[137] Parolin C., Frisco G., Foschi C., Giordani B., Salvo M., Vitali B., Marangoni A., Calonghi N. (2018). *Lactobacillus crispatus* BC5 Interferes with *Chlamydia trachomatis* Infectivity through Integrin Modulation in Cervical Cells. Front. Microbiol..

[138] Kaewsrichan J., Peeyananjarassri K., Kongprasertkit J. (2006). Selection and identification of anaerobic lactobacilli producing inhibitory compounds against vaginal pathogens. FEMS Immunol. Med. Microbiol..

[139] Shavandi A., Saeedi P., Gérard P., Jalalvandi E., Cannella D., Bekhit A.E.-D. (2020). The role of microbiota in tissue repair and regeneration. J. Tissue Eng. Regen. Med..

[140] Alam A., Neish A. (2018). Role of gut microbiota in intestinal wound healing and barrier function. Tissue Barriers.

[141] Stefan K.L., Kim M.V., Iwasaki A., Kasper D.L. (2020). Commensal Microbiota Modulation of Natural Resistance to Virus Infection. Cell.

[142] Pang I.K., Iwasaki A. (2012). Control of antiviral immunity by pattern recognition and the microbiome. Immunol. Rev..

[143] Rizzello V., Bonaccorsi I., Dongarrà M.L., Fink L.N., Ferlazzo G. (2011). Role of natural killer and dendritic cell crosstalk in immunomodulation by commensal bacteria probiotics. J. Biomed. Biotechnol..

[144] Karst S.M. (2016). The influence of commensal bacteria on infection with enteric viruses. Nat. Rev. Microbiol..

[145] Wilks J., Beilinson H., Golovkina T.V. (2013). Dual role of commensal bacteria in viral infections. Immunol. Rev..

[146] Li N., Ma W.-T., Pang M., Fan Q.-L., Hua J.-L. (2019). The Commensal Microbiota and Viral Infection: A Comprehensive Review. Front. Immunol..

[147] Wilks J., Golovkina T. (2012). Influence of microbiota on viral infections. PLoS Pathog..

[148] Sharma R., Young C., Neu J. (2010). Molecular modulation of intestinal epithelial barrier: Contribution of microbiota. J. Biomed. Biotechnol..

[149] Fakharian F., Thirugnanam S., Welsh D.A., Kim W.-K., Rappaport J., Bittinger K., Rout N. (2023). The Role of Gut Dysbiosis in the Loss of Intestinal Immune Cell Functions and Viral Pathogenesis. Microorganisms.

[150] Boulangé C.L., Neves A.L., Chilloux J., Nicholson J.K., Dumas M.-E. (2016). Impact of the gut microbiota on inflammation, obesity, and metabolic disease. Genome Med..

[151] Newman N.K., Zhang Y., Padiadpu J., Miranda C.L., Magana A.A., Wong C.P., Hioki K.A., Pederson J.W., Li Z., Gurung M. (2023). Reducing gut microbiome-driven adipose tissue inflammation alleviates metabolic syndrome. Microbiome.

[152] Zeng M.Y., Inohara N., Nuñez G. (2017). Mechanisms of inflammation-driven bacterial dysbiosis in the gut. Mucosal Immunol..

[153] van den Elsen L.W., Poyntz H.C., Weyrich L.S., Young W., Forbes-Blom E.E. (2017). Embracing the gut microbiota: The new frontier for inflammatory and infectious diseases. Clin. Transl. Immunol..

[154] Fujio-Vejar S., Vasquez Y., Morales P., Magne F., Vera-Wolf P., Ugalde J.A., Navarrete P., Gotteland M. (2017). The Gut Microbiota of Healthy Chilean Subjects Reveals a High Abundance of the Phylum *Verrucomicrobia*. Front. Microbiol..

[155] Zilberman-Schapira G., Zmora N., Itav S., Bashiardes S., Elinav H., Elinav E. (2016). The gut microbiome in human immunodeficiency virus infection. BMC Med..

[156] Berbers R.-M., Nierkens S., van Laar J.M., Bogaert D., Leavis H.L. (2017). Microbial Dysbiosis in Common Variable Immune Deficiencies: Evidence, Causes, and Consequences. Trends Immunol..

[157] Pellicciotta M., Rigoni R., Falcone E.L., Holland S.M., Villa A., Cassani B. (2019). The microbiome and immunodeficiencies: Lessons from rare diseases. J. Autoimmun..

[158] Khosravi A., Mazmanian S.K. (2013). Disruption of the gut microbiome as a risk factor for microbial infections. Curr. Opin. Microbiol..

[159] Luo A., Leach S.T., Barres R., Hesson L.B., Grimm M.C., Simar D. (2017). The Microbiota and Epigenetic Regulation of T Helper 17/Regulatory T Cells: In Search of a Balanced Immune System. Front. Immunol..

[160] Esposito P., Ismail N. (2022). Linking Puberty and the Gut Microbiome to the Pathogenesis of Neurodegenerative Disorders. Microorganisms.

[161] Rusch J.A., Layden B.T., Dugas L.R. (2023). Signalling cognition: The gut microbiota and hypothalamic-pituitary-adrenal axis. Front. Endocrinol..

[162] Castellazzi A., Tagliacarne S.C., Soldi S., Perna S., Ziviani L., Milleri S., Montagna L., Valsecchi C. (2018). Stress and Immune Function: There is a Role for the Gut Microbiota?. J. Clin. Gastroenterol..

[163] Lyte M. (2016). Microbial Endocrinology in the Pathogenesis of Infectious Disease. Virulence Mechanisms of Bacterial Pathogens.

[164] Sarkodie E.K., Zhou S., Baidoo S.A., Chu W. (2019). Influences of stress hormones on microbial infections. Microb. Pathog..

[165] Catalkaya G., Venema K., Lucini L., Rocchetti G., Delmas D., Daglia M., de Filippis A., Xiao H., Quiles J.L., Xiao J. (2020). Interaction of dietary polyphenols and gut microbiota: Microbial metabolism of polyphenols, influence on the gut microbiota, and implications on host health. Food Front..

[166] Wang G., Huang S., Wang Y., Cai S., Yu H., Liu H., Zeng X., Zhang G., Qiao S. (2019). Bridging intestinal immunity and gut microbiota by metabolites. Cell. Mol. Life Sci..

[167] Diamond G., Beckloff N., Weinberg A., Kisich K.O. (2009). The Roles of Antimicrobial Peptides in Innate Host Defense.

[168] Cole J.N., Nizet V. (2016). Bacterial Evasion of Host Antimicrobial Peptide Defenses. Virulence Mechanisms of Bacterial Pathogens.

[169] Hancock R.E.W., Haney E.F., Gill E.E. (2016). The immunology of host defence peptides: Beyond antimicrobial activity. Nat. Rev. Immunol..

[170] Ribeiro C.F.A., Silveira G.G.D.O.S., Candido E.D.S., Cardoso M.H., Espinola Carvalho C.M., Franco O.L. (2020). Effects of Antibiotic Treatment on Gut Microbiota and How to Overcome Its Negative Impacts on Human Health. ACS Infect. Dis..

[171] Langdon A., Crook N., Dantas G. (2016). The effects of antibiotics on the microbiome throughout development and alternative approaches for therapeutic modulation. Genome Med..

[172] Lambring C.B., Siraj S., Patel K., Sankpal U.T., Mathew S., Basha R. (2019). Impact of the Microbiome on the Immune System. Crit. Rev. Immunol..

[173] Feng W., Liu J., Ao H., Yue S., Peng C. (2020). Targeting gut microbiota for precision medicine: Focusing on the efficacy and toxicity of drugs. Theranostics.

[174] Yang M., Yang Y., He Q., Zhu P., Liu M., Xu J., Zhao M. (2021). Intestinal Microbiota-A Promising Target for Antiviral Therapy?. Front. Immunol..

[175] Zeng Y., Chen S., Fu Y., Wu W., Chen T., Chen J., Yang B., Ou Q. (2020). Gut microbiota dysbiosis in patients with hepatitis B virus-induced chronic liver disease covering chronic hepatitis, liver cirrhosis and hepatocellular carcinoma. J. Viral Hepat..

[176] Ren Y.-D., Ye Z.-S., Yang L.-Z., Jin L.-X., Wei W.-J., Deng Y.-Y., Chen X.-X., Xiao C.-X., Yu X.-F., Xu H.-Z. (2017). Fecal microbiota transplantation induces hepatitis B virus e-antigen (HBeAg) clearance in patients with positive HBeAg after long-term antiviral therapy. Hepatology.

[177] Steiner H.E., Patterson H.K., Giles J.B., Karnes J.H. (2022). Bringing pharmacomicrobiomics to the clinic through well-designed studies. Clin. Transl. Sci..

[178] Li Y.-N., Kang N.-L., Jiang J.-J., Zhu Y.-Y., Liu Y.-R., Zeng D.-W., Wang F. (2022). Gut microbiota of hepatitis B virus-infected patients in the immune-tolerant and immune-active phases and their implications in metabolite changes. World J. Gastroenterol..

[179] Li Y.-G., Yu Z.-J., Li A., Ren Z.-G. (2022). Gut microbiota alteration and modulation in hepatitis B virus-related fibrosis and complications: Molecular mechanisms and therapeutic inventions. World J. Gastroenterol..

[180] Gu L., Deng H., Ren Z., Zhao Y., Yu S., Guo Y., Dai J., Chen X., Li K., Li R. (2019). Dynamic Changes in the Microbiome and Mucosal Immune Microenvironment of the Lower Respiratory Tract by Influenza Virus Infection. Front. Microbiol..

[181] Hanada S., Pirzadeh M., Carver K.Y., Deng J.C. (2018). Respiratory Viral Infection-Induced Microbiome Alterations and Secondary Bacterial Pneumonia. Front. Immunol..

[182] Nhu N.T.Q., Young V.B. (2023). The Relationship Between the Microbiome and Antimicrobial Resistance. Clin. Infect. Dis..

[183] McInnes R.S., McCallum G.E., Lamberte L.E., van Schaik W. (2020). Horizontal transfer of antibiotic resistance genes in the human gut microbiome. Curr. Opin. Microbiol..

[184] Modi S.R., Collins J.J., Relman D.A. (2014). Antibiotics and the gut microbiota. J. Clin. Investig..

[185] Culligan E.P., Hill C., Sleator R.D. (2009). Probiotics and gastrointestinal disease: Successes, problems and future prospects. Gut Pathog..

[186] Borchert D., Sheridan L., Papatsoris A., Faruquz Z., Barua J.M., Junaid I., Pati Y., Chinegwundoh F., Buchholz N. (2008). Prevention and treatment of urinary tract infection with probiotics: Review and research perspective. Indian J. Urol. IJU J. Urol. Soc. India.

[187] Yan F., Polk D.B. (2006). Probiotics as functional food in the treatment of diarrhea. Curr. Opin. Clin. Nutr. Metab. Care.

[188] Lozupone C.A. (2018). Unraveling Interactions between the Microbiome and the Host Immune System to Decipher Mechanisms of Disease. mSystems.

[189] Zimmermann M., Patil K.R., Typas A., Maier L. (2021). Towards a mechanistic understanding of reciprocal drug-microbiome interactions. Mol. Syst. Biol..

[190] Alexander J.L., Wilson I.D., Teare J., Marchesi J.R., Nicholson J.K., Kinross J.M. (2017). Gut microbiota modulation of chemotherapy efficacy and toxicity. Nat. Rev. Gastroenterol. Hepatol..

[191] Zimmermann M., Zimmermann-Kogadeeva M., Wegmann R., Goodman A.L. (2019). Mapping human microbiome drug metabolism by gut bacteria and their genes. Nature.

[192] Pant A., Maiti T.K., Mahajan D., Das B. (2023). Human Gut Microbiota and Drug Metabolism. Microb. Ecol..

[193] Wilson I.D., Nicholson J.K. (2009). The Role of Gut Microbiota in Drug Response.

[194] Sousa T., Paterson R., Moore V., Carlsson A., Abrahamsson B., Basit A.W. (2008). The gastrointestinal microbiota as a site for the biotransformation of drugs. Int. J. Pharm..

[195] Iliev I.D., Leonardi I. (2017). Fungal dysbiosis: Immunity and interactions at mucosal barriers. Nat. Rev. Immunol..

[196] Roudbary M., Kumar S., Kumar A., Černáková L., Nikoomanesh F., Rodrigues C.F. (2021). Overview on the Prevalence of Fungal Infections, Immune Response, and Microbiome Role in COVID-19 Patients. J. Fungi.

[197] Patel P., Poudel A., Kafle S., Thapa Magar M., Cancarevic I. (2021). Influence of Microbiome and Antibiotics on the Efficacy of Immune Checkpoint Inhibitors. Cureus.

[198] Zhang W., Zhang Y., Li Y., Ma D., Zhang H., Kwok L. (2022). *Lacticaseibacillus rhamnosus* Probio-M9-Driven Mouse Mammary Tumor-Inhibitory Effect Is Accompanied by Modulation of Host Gut Microbiota, Immunity, and Serum Metabolome. Nutrients.

[199] Benson A.K., Kelly S.A., Legge R., Ma F., Low S.J., Kim J., Zhang M., Oh P.L., Nehrenberg D., Hua K. (2010). Individuality in gut microbiota composition is a complex polygenic trait shaped by multiple environmental and host genetic factors. Proc. Natl. Acad. Sci. USA.

[200] Wu G.D., Chen J., Hoffmann C., Bittinger K., Chen Y.-Y., Keilbaugh S.A., Bewtra M., Knights D., Walters W.A., Knight R. (2011). Linking long-term dietary patterns with gut microbial enterotypes. Science.

[201] Bibbò S., Ianiro G., Giorgio V., Scaldaferri F., Masucci L., Gasbarrini A., Cammarota G. (2016). The role of diet on gut microbiota composition. Eur. Rev. Med. Pharmacol. Sci..

[202] Tomova A., Bukovsky I., Rembert E., Yonas W., Alwarith J., Barnard N.D., Kahleova H. (2019). The Effects of Vegetarian and Vegan Diets on Gut Microbiota. Front. Nutr..

[203] Merra G., Noce A., Marrone G., Cintoni M., Tarsitano M.G., Capacci A., de Lorenzo A. (2020). Influence of Mediterranean Diet on Human Gut Microbiota. Nutrients.

[204] Deledda A., Palmas V., Heidrich V., Fosci M., Lombardo M., Cambarau G., Lai A., Melis M., Loi E., Loviselli A. (2022). Dynamics of Gut Microbiota and Clinical Variables after Ketogenic and Mediterranean Diets in Drug-Naïve Patients with Type 2 Diabetes Mellitus and Obesity. Metabolites.

[205] Willmann M., Peter S. (2017). Translational metagenomics and the human resistome: Confronting the menace of the new millennium. J. Mol. Med..

[206] Reens A.L., Cabral D.J., Liang X., Norton J.E., Therien A.G., Hazuda D.J., Swaminathan G. (2021). Immunomodulation by the Commensal Microbiome During Immune-Targeted Interventions: Focus on Cancer Immune Checkpoint Inhibitor Therapy and Vaccination. Front. Immunol..

[207] de Jong S.E., Olin A., Pulendran B. (2020). The Impact of the Microbiome on Immunity to Vaccination in Humans. Cell Host Microbe.

[208] Valdez Y., Brown E.M., Finlay B.B. (2014). Influence of the microbiota on vaccine effectiveness. Trends Immunol..

[209] Yang R., Chen Z., Cai J. (2023). Fecal microbiota transplantation: Emerging applications in autoimmune diseases. J. Autoimmun..

[210] Waller K.M.J., Leong R.W., Paramsothy S. (2022). An update on fecal microbiota transplantation for the treatment of gastrointestinal diseases. J. Gastroenterol. Hepatol..

[211] Pickard J.M., Zeng M.Y., Caruso R., Núñez G. (2017). Gut microbiota: Role in pathogen colonization, immune responses, and inflammatory disease. Immunol. Rev..

[212] Bäumler A.J., Sperandio V. (2016). Interactions between the microbiota and pathogenic bacteria in the gut. Nature.

[213] Wiegers C., van de Burgwal L.H.M., Larsen O.F.A. (2022). Probiotics for the Management of Infectious Diseases: Reviewing the State of the Art. Front. Microbiol..

[214] Morgan X.C., Huttenhower C. (2012). Chapter 12: Human microbiome analysis. PLoS Comput. Biol..

[215] Kumar R., Yadav G., Kuddus M., Ashraf G.M., Singh R. (2023). Unlocking the microbial studies through computational approaches: How far have we reached?. Environ. Sci. Pollut. Res..

[216] Galloway-Peña J., Hanson B. (2020). Tools for Analysis of the Microbiome. Dig. Dis. Sci..

[217] Spalinger M.R., Scharl M. (2023). Microbiota Manipulation as an Emerging Concept in Cancer Therapy. Visc. Med..

[218] Zhang M., Liu J., Xia Q. (2023). Role of gut microbiome in cancer immunotherapy: From predictive biomarker to therapeutic target. Exp. Hematol. Oncol..

[219] Cheng W.Y., Wu C.-Y., Yu J. (2020). The role of gut microbiota in cancer treatment: Friend or foe?. Gut.

[220] Nguyen D.-H., Chong A., Hong Y., Min J.-J. (2023). Bioengineering of bacteria for cancer immunotherapy. Nat. Commun..

[221] Shahbaz A., Mahmood T., Javed M.U., Abbasi B.H. (2023). Current advances in microbial-based cancer therapies. Med. Oncol..

[222] King A.M., Zhang Z., Glassey E., Siuti P., Clardy J., Voigt C.A. (2023). Systematic mining of the human microbiome identifies antimicrobial peptides with diverse activity spectra. Nat. Microbiol..

[223] Li X., Wang Q., Hu X., Liu W. (2022). Current Status of Probiotics as Supplements in the Prevention and Treatment of Infectious Diseases. Front. Cell. Infect. Microbiol..

[224] Monye I., Adelowo A.B. (2020). Strengthening immunity through healthy lifestyle practices: Recommendations for lifestyle interventions in the management of COVID-19. Lifestyle Med..

[225] Wastyk H.C., Fragiadakis G.K., Perelman D., Dahan D., Merrill B.D., Yu F.B., Topf M., Gonzalez C.G., van Treuren W., Han S. (2021). Gut-microbiota-targeted diets modulate human immune status. Cell.

[226] Zhang Y., Dong A., Xie K., Yu Y. (2018). Dietary Supplementation with High Fiber Alleviates Oxidative Stress and Inflammatory Responses Caused by Severe Sepsis in Mice Without Altering Microbiome Diversity. Front. Physiol..

[227] Sathiananthamoorthy S., Florman K., Richard D., Cheng K.K., Torri V., McCaig F., Harber M., Rohn J.L. (2023). Application of Various Techniques to Gain Insights into the Complex Urinary Tract Microbial Communities of Renal Transplant Recipients. Transplant. Direct.

[228] Bustamante M., Oomah B.D., Oliveira W.P., Burgos-Díaz C., Rubilar M., Shene C. (2020). Probiotics and prebiotics potential for the care of skin, female urogenital tract, and respiratory tract. Folia Microbiol..

[229] Holmes E., Li J.V., Athanasiou T., Ashrafian H., Nicholson J.K. (2011). Understanding the role of gut microbiome-host metabolic signal disruption in health and disease. Trends Microbiol..

[230] Gomaa E.Z. (2020). Human gut microbiota/microbiome in health and diseases: A review. Antonie Van Leeuwenhoek.

[231] Zhang Y., Lun C.-Y., Tsui S.K.-W. (2015). Metagenomics: A New Way to Illustrate the Crosstalk between Infectious Diseases and Host Microbiome. Int. J. Mol. Sci..

[232] Bandera A., de Benedetto I., Bozzi G., Gori A. (2018). Altered gut microbiome composition in HIV infection: Causes, effects and potential intervention. Curr. Opin. HIV AIDS.

[233] Lozupone C.A., Li M., Campbell T.B., Flores S.C., Linderman D., Gebert M.J., Knight R., Fontenot A.P., Palmer B.E. (2013). Alterations in the gut microbiota associated with HIV-1 infection. Cell Host Microbe.

[234] Chen P., He G., Qian J., Zhan Y., Xiao R. (2021). Potential role of the skin microbiota in Inflammatory skin diseases. J. Cosmet. Dermatol..

[235] Nørreslet L.B., Agner T., Clausen M.-L. (2020). The Skin Microbiome in Inflammatory Skin Diseases. Curr. Dermatol. Rep..

[236] Nicholas-Haizelden K., Murphy B., Hoptroff M., Horsburgh M.J. (2023). Bioprospecting the Skin Microbiome: Advances in Therapeutics and Personal Care Products. Microorganisms.

[237] AL-Smadi K., Leite-Silva V.R., Filho N.A., Lopes P.S., Mohammed Y. (2023). Innovative Approaches for Maintaining and Enhancing Skin Health and Managing Skin Diseases through Microbiome-Targeted Strategies. Antibiotics.

[238] Zhou H., Shi L., Ren Y., Tan X., Liu W., Liu Z. (2020). Applications of Human Skin Microbiota in the Cutaneous Disorders for Ecology-Based Therapy. Front. Cell. Infect. Microbiol..

[239] McDonald D., Ackermann G., Khailova L., Baird C., Heyland D., Kozar R., Lemieux M., Derenski K., King J., Vis-Kampen C. (2016). Extreme Dysbiosis of the Microbiome in Critical Illness. mSphere.

[240] Lankelma J.M., Cranendonk D.R., Belzer C., de Vos A.F., de Vos W.M., van der Poll T., Wiersinga W.J. (2017). Antibiotic-induced gut microbiota disruption during human endotoxemia: A randomised controlled study. Gut.

[241] Putignani L. (2012). Human gut microbiota: Onset and shaping through life stages and perturbations. Front. Cell. Infect. Microbiol..

[242] Palmer C., Bik E.M., DiGiulio D.B., Relman D.A., Brown P.O. (2007). Development of the human infant intestinal microbiota. PLoS Biol..

[243] Petschow B., Doré J., Hibberd P., Dinan T., Reid G., Blaser M., Cani P.D., Degnan F.H., Foster J., Gibson G. (2013). Probiotics, prebiotics, and the host microbiome: The science of translation. Ann. N. Y. Acad. Sci..

[244] Jernberg C., Löfmark S., Edlund C., Jansson J.K. (2010). Long-term impacts of antibiotic exposure on the human intestinal microbiota. Microbiology.

[245] Yassour M., Vatanen T., Siljander H., Hämäläinen A.-M., Härkönen T., Ryhänen S.J., Franzosa E.A., Vlamakis H., Huttenhower C., Gevers D. (2016). Natural history of the infant gut microbiome and impact of antibiotic treatment on bacterial strain diversity and stability. Sci. Transl. Med..

[246] Kent A.G., Vill A.C., Shi Q., Satlin M.J., Brito I.L. (2020). Widespread transfer of mobile antibiotic resistance genes within individual gut microbiomes revealed through bacterial Hi-C. Nat. Commun..

[247] Zuo T., Liang G., Huang Z., Cao Z., Bai F., Zhou Y., Wu X., Wu X., Chen Y.-Q., Balati M. (2023). Baseline gut microbiome features prior to SARS-CoV-2 infection are associated with host symptoms in and post COVID-19. J. Med. Virol..

[248] de Filippo C., Cavalieri D., Di Paola M., Ramazzotti M., Poullet J.B., Massart S., Collini S., Pieraccini G., Lionetti P. (2010). Impact of diet in shaping gut microbiota revealed by a comparative study in children from Europe and rural Africa. Proc. Natl. Acad. Sci. USA.

[249] Alvarez-Olmos M.I., Oberhelman R.A. (2001). Probiotic agents and infectious diseases: A modern perspective on a traditional therapy. Clin. Infect. Dis..

[250] Mamo Y., Woodworth M., Sitchenko K., Dhere T., Kraft C. (2017). Durability and Long-Term Clinical Outcomes of Fecal Microbiota Transplant (FMT) Treatment in Patients with Recurrent C. difficile Infection. Open Forum Infect. Dis..

[251] Dowle C. (2016). Faecal microbiota transplantation: A review of FMT as an alternative treatment for Clostridium difficile infection. Biosci. Horiz..

[252] Łusiak-Szelachowska M., Międzybrodzki R., Drulis-Kawa Z., Cater K., Knežević P., Winogradow C., Amaro K., Jończyk-Matysiak E., Weber-Dąbrowska B., Rękas J. (2022). Bacteriophages and antibiotic interactions in clinical practice: What we have learned so far. J. Biomed. Sci..

[253] Romero-Calle D., Guimarães Benevides R., Góes-Neto A., Billington C. (2019). Bacteriophages as Alternatives to Antibiotics in Clinical Care. Antibiotics.

[254] Curtis M.A., Zenobia C., Darveau R.P. (2011). The relationship of the oral microbiotia to periodontal health and disease. Cell Host Microbe.

[255] Zhu X., Yan S., Yuan F., Wan S. (2020). The Applications of Nanopore Sequencing Technology in Pathogenic Microorganism Detection. Can. J. Infect. Dis. Med. Microbiol..

[256] Maier L., Pruteanu M., Kuhn M., Zeller G., Telzerow A., Anderson E.E., Brochado A.R., Fernandez K.C., Dose H., Mori H. (2018). Extensive impact of non-antibiotic drugs on human gut bacteria. Nature.

[257] Mantziari A., Salminen S., Szajewska H., Malagón-Rojas J.N. (2020). Postbiotics against Pathogens Commonly Involved in Pediatric Infectious Diseases. Microorganisms.

[258] Ma L., Tu H., Chen T. (2023). Postbiotics in Human Health: A Narrative Review. Nutrients.

[259] Żółkiewicz J., Marzec A., Ruszczyński M., Feleszko W. (2020). Postbiotics-A Step Beyond Pre- and Probiotics. Nutrients.

[260] Mazhar S.F., Afzal M., Almatroudi A., Munir S., Ashfaq U.A., Rasool M., Raza H., Munir H.M.W., Rajoka M.S.R., Khurshid M. (2020). The Prospects for the Therapeutic Implications of Genetically Engineered Probiotics. J. Food Qual..

[261] Zhou Z., Chen X., Sheng H., Shen X., Sun X., Yan Y., Wang J., Yuan Q. (2020). Engineering probiotics as living diagnostics and therapeutics for improving human health. Microb. Cell Fact..

[262] Aparna Y., Anuradha K., Anju S., Sarada J. (2021). A Systematic Review on Crosstalk Between Microbiome and Immune System. Microbiome in Human Health and Disease.

[263] Górski A., Międzybrodzki R., Weber-Dąbrowska B., Fortuna W., Letkiewicz S., Rogóż P., Jończyk-Matysiak E., Dąbrowska K., Majewska J., Borysowski J. (2016). Phage Therapy: Combating Infections with Potential for Evolving from Merely a Treatment for Complications to Targeting Diseases. Front. Microbiol..

[264] Golkar Z., Bagasra O., Pace D.G. (2014). Bacteriophage therapy: A potential solution for the antibiotic resistance crisis. J. Infect. Dev. Ctries..

[265] Moelling K., Broecker F., Willy C. (2018). A Wake-Up Call: We Need Phage Therapy Now. Viruses.

[266] Abedon S., Danis-Wlodarczyk K., Alves D. (2021). Phage Therapy in the 21st Century: Is There Modern, Clinical Evidence of Phage-Mediated Efficacy?. Pharmaceuticals.

[267] Chang C., Yu X., Guo W., Guo C., Guo X., Li Q., Zhu Y. (2022). Bacteriophage-Mediated Control of Biofilm: A Promising New Dawn for the Future. Front. Microbiol..

[268] Tian F., Li J., Nazir A., Tong Y. (2021). Bacteriophage—A Promising Alternative Measure for Bacterial Biofilm Control. Infect. Drug Resist..

[269] Hitch T., Hall L., Walsh S.K., Leventhal G., Slack E., de Wouters T., Walter J., Clavel T. (2022). Microbiome-based interventions to modulate gut ecology and the immune system. Mucosal Immunol..

[270] Choy A., Freedberg D.E. (2020). Impact of microbiome-based interventions on gastrointestinal pathogen colonization in the intensive care unit. Ther. Adv. Gastroenterol..

[271] Saini K., Minj J. (2020). Multifunctional Aspects of Probiotics and Prebiotics in Health Management: An Overview. Dairy Processing: Advanced Research to Applications.

[272] Petrov Ivanković A., Veljković M., Ćorović M., Milivojević A., Simović M., Banjanac K., Bezbradica D. Evaluation of Herbal Extracts for Their Potential Application as Skin Prebiotics. Proceedings of the 16th International Conference on Advances in Probiotics and Prebiotics.

[273] Fox B.E., Vilander A.C., Gilfillan D., Dean G.A., Abdo Z. (2022). Oral Vaccination Using a Probiotic Vaccine Platform Combined with Prebiotics Impacts Immune Response and the Microbiome. Vaccines.

[274] Gezginç Y., Karabekmez-Erdem T., Tatar H.D., Ayman S., Ganiyusufoğlu E., Dayisoylu K.S. (2022). Health promoting benefits of postbiotics produced by lactic acid bacteria: Exopolysaccharide. Biotech Stud..

[275] Vera-Santander V.E., Hernández-Figueroa R.H., Jiménez-Munguía M.T., Mani-López E., López-Malo A. (2023). Health Benefits of Consuming Foods with Bacterial Probiotics, Postbiotics, and Their Metabolites: A Review. Molecules.

[276] Scott E., de Paepe K., van de Wiele T. (2022). Postbiotics and Their Health Modulatory Biomolecules. Biomolecules.

[277] Suluvoy J.K., Gomez P.L., Joel T., Toppo N., Karthikeyan D., Shepherd R. (2021). Nanoparticles as Antimicrobial Agents and Drug Delivery Systems—A Review. J. Pure Appl. Microbiol..

[278] Fadaka A.O., Sibuyi N.R.S., Madiehe A.M., Meyer M. (2021). Nanotechnology-Based Delivery Systems for Antimicrobial Peptides. Pharmaceutics.

[279] Mercan D.-A., Niculescu A.-G., Grumezescu A.M. (2022). Nanoparticles for Antimicrobial Agents Delivery-An Up-to-Date Review. Int. J. Mol. Sci..

[280] Nigam A., Kalauni K., Pawar S.J. (2022). Physio-chemical characterizations and antimicrobial properties of nano-sized Mg-Zn ferrite particles for biomedical applications. Mater. Technol..

[281] Katyal D., Jain R.K., Sreenivasagan S. (2022). Herbal-mediated preparation of nano-sized particles of selenium, its characterization, and evaluation of its antimicrobial activity. J. Adv. Pharm. Technol. Res..

[282] Liew K.B., Janakiraman A.K., Sundarapandian R., Khalid S.H., Razzaq F.A., Ming L.C., Khan A., Kalusalingam A., Ng P.W. (2022). A review and revisit of nanoparticles for antimicrobial drug delivery. J. Med. Life.

[283] Yuan C., He Y., Xie K., Feng L., Gao S., Cai L. (2023). Review of microbiota gut brain axis and innate immunity in inflammatory and infective diseases. Front. Cell. Infect. Microbiol..

[284] Zhu X., Han Y., Du J., Liu R., Jin K., Yi W. (2017). Microbiota-gut-brain axis and the central nervous system. Oncotarget.

[285] Borenstein E. (2012). Computational systems biology and in silico modeling of the human microbiome. Brief. Bioinform..

[286] Gevers D., Pop M., Schloss P.D., Huttenhower C. (2012). Bioinformatics for the Human Microbiome Project. PLoS Comput. Biol..

[287] Kazemifard N., Dehkohneh A., Baradaran Ghavami S. (2022). Probiotics and probiotic-based vaccines: A novel approach for improving vaccine efficacy. Front. Med..

[288] Pfalzgraff A., Brandenburg K., Weindl G. (2018). Antimicrobial Peptides and Their Therapeutic Potential for Bacterial Skin Infections and Wounds. Front. Pharmacol..

[289] Luong H.X., Thanh T.T., Tran T.H. (2020). Antimicrobial peptides—Advances in development of therapeutic applications. Life Sci..

[290] Hollmann A., Martinez M., Maturana P., Semorile L.C., Maffia P.C. (2018). Antimicrobial Peptides: Interaction with Model and Biological Membranes and Synergism with Chemical Antibiotics. Front. Chem..

[291] Zhang Q.-Y., Yan Z.-B., Meng Y.-M., Hong X.-Y., Shao G., Ma J.-J., Cheng X.-R., Liu J., Kang J., Fu C.-Y. (2021). Antimicrobial peptides: Mechanism of action, activity and clinical potential. Mil. Med. Res..

[292] Deshayes C., Arafath M.N., Apaire-Marchais V., Roger E. (2021). Drug Delivery Systems for the Oral Administration of Antimicrobial Peptides: Promising Tools to Treat Infectious Diseases. Front. Med. Technol..

[293] Shaw L.P., Bassam H., Barnes C.P., Walker A.S., Klein N., Balloux F. (2019). Modelling microbiome recovery after antibiotics using a stability landscape framework. ISME J..

[294] Ramachandran G., Bikard D. (2019). Editing the microbiome the CRISPR way. Philos. Trans. R. Soc. Lond. B Biol. Sci..

[295] Bhattacharyya S., Mukherjee A. (2020). CRISPR: The Revolutionary Gene Editing Tool with Far-Reaching Applications. Biotechnology Business-Concept to Delivery.

[296] Zhang D., Hussain A., Manghwar H., Xie K., Xie S., Zhao S., Larkin R.M., Qing P., Jin S., Ding F. (2020). Genome editing with the CRISPR-Cas system: An art, ethics and global regulatory perspective. Plant Biotechnol. J..

[297] Lee Y., Kamada N., Moon J.J. (2021). Oral nanomedicine for modulating immunity, intestinal barrier functions, and gut microbiome. Adv. Drug Deliv. Rev..

[298] El-Sayed A., Kamel M. (2020). Advances in nanomedical applications: Diagnostic, therapeutic, immunization, and vaccine production. Environ. Sci. Pollut. Res..

[299] Fatima F., Siddiqui S., Khan W.A. (2021). Nanoparticles as Novel Emerging Therapeutic Antibacterial Agents in the Antibiotics Resistant Era. Biol. Trace Elem. Res..

[300] Adeniji O.O., Nontongana N., Okoh J.C., Okoh A.I. (2022). The Potential of Antibiotics and Nanomaterial Combinations as Therapeutic Strategies in the Management of Multidrug-Resistant Infections: A Review. Int. J. Mol. Sci..

[301] Garg P., Attri P., Sharma R., Chauhan M., Chaudhary G.R. (2022). Advances and Perspective on Antimicrobial Nanomaterials for Biomedical Applications. Front. Nanotechnol..

[302] El-Sayed A., Kamel M. (2020). Advanced applications of nanotechnology in veterinary medicine. Environ. Sci. Pollut. Res..

[303] Biswas P., Polash S.A., Dey D., Kaium M.A., Mahmud A.R., Yasmin F., Baral S.K., Islam M.A., Rahaman T.I., Abdullah A. (2023). Advanced implications of nanotechnology in disease control and environmental perspectives. Biomed. Pharmacother..

[304] Arun K.B., Sindhu R., Alex D., Binod P., Pughazhendi A., Joseph T.C., Pandey A., Kuddus M., Pillai S., Emmanual S. (2022). Bacterial bioactive metabolites as therapeutic agents: From production to action. Sustain. Chem. Pharm..

[305] Rafique N., Jan S.Y., Dar A.H., Dash K.K., Sarkar A., Shams R., Pandey V.K., Khan S.A., Amin Q.A., Hussain S.Z. (2023). Promising bioactivities of postbiotics: A comprehensive review. J. Agric. Food Res..

[306] Sorbara M.T., Pamer E.G. (2022). Microbiome-based therapeutics. Nat. Rev. Microbiol..

[307] Mimee M., Citorik R.J., Lu T.K. (2016). Microbiome therapeutics—Advances and challenges. Adv. Drug Deliv. Rev..

[308] Pham J.V., Yilma M.A., Feliz A., Majid M.T., Maffetone N., Walker J.R., Kim E., Cho H.J., Reynolds J.M., Song M.C. (2019). A Review of the Microbial Production of Bioactive Natural Products and Biologics. Front. Microbiol..

[309] Mabwi H.A., Kim E., Song D.-G., Yoon H.S., Pan C.-H., Komba E.V.G., Ko G., Cha K.H. (2021). Synthetic gut microbiome: Advances and challenges. Comput. Struct. Biotechnol. J..

[310] Duncker K.E., Holmes Z.A., You L. (2021). Engineered microbial consortia: Strategies and applications. Microb. Cell Fact..

[311] Simons A., Alhanout K., Duval R.E. (2020). Bacteriocins, Antimicrobial Peptides from Bacterial Origin: Overview of Their Biology and Their Impact against Multidrug-Resistant Bacteria. Microorganisms.

[312] Ołdak A., Zielińska D. (2017). Bacteriocins from lactic acid bacteria as an alternative to antibiotics. Postep. Hig. Med. Dosw..

[313] Twomey E., Hill C., Field D., Begley M. (2021). Recipe for Success: Suggestions and Recommendations for the Isolation and Characterisation of Bacteriocins. Int. J. Microbiol..

[314] Cotter P.D., Ross R.P., Hill C. (2013). Bacteriocins—A viable alternative to antibiotics?. Nat. Rev. Microbiol..

[315] Bokoliya S.C., Dorsett Y., Panier H., Zhou Y. (2021). Procedures for Fecal Microbiota Transplantation in Murine Microbiome Studies. Front. Cell. Infect. Microbiol..

[316] Bosco N., Noti M. (2021). The aging gut microbiome and its impact on host immunity. Genes. Immun..

[317] Ezzamouri B., Shoaie S., Ledesma-Amaro R. (2021). Synergies of Systems Biology and Synthetic Biology in Human Microbiome Studies. Front. Microbiol..

[318] Mousavinasab F., Karimi R., Taheri S., Ahmadvand F., Sanaaee S., Najafi S., Halvaii M.S., Haghgoo A., Zamany M., Majidpoor J. (2023). Microbiome modulation in inflammatory diseases: Progress to microbiome genetic engineering. Cancer Cell Int..

[319] Kashyap P.C., Chia N., Nelson H., Segal E., Elinav E. (2017). Microbiome at the Frontier of Personalized Medicine. Mayo Clin. Proc..

[320] Yi X., Lu H., Liu X., He J., Li B., Wang Z., Zhao Y., Zhang X., Yu X. (2023). Unravelling the enigma of the human microbiome: Evolution and selection of sequencing technologies. Microb. Biotechnol..

[321] Parodi G., Leite G., Pimentel M.L., Barlow G.M., Fiorentino A., Morales W., Pimentel M., Weitsman S., Mathur R. (2022). The Response of the Rodent Gut Microbiome to Broad-Spectrum Antibiotics Is Different in Males and Females. Front. Microbiol..

[322] McGovern B., Sims M., Kraft C., Wang E., Brady K., Ford C., Edward O., Lombardo M.-J., Wortman J., Litcofsky K. (2021). Efficacy and Safety of Investigational Microbiome Drug SER-109 for Treatment of Recurrent *Clostridioides difficile* Infection. Antimicrob. Steward. Healthc. Epidemiol..

[323] Ratiner K., Ciocan D., Abdeen S.K., Elinav E. (2023). Utilization of the microbiome in personalized medicine. Nat. Rev. Microbiol..

[324] Maciel-Fiuza M.F., Muller G.C., Campos D.M.S., do Socorro Silva Costa P., Peruzzo J., Bonamigo R.R., Veit T., Vianna F.S.L. (2023). Role of gut microbiota in infectious and inflammatory diseases. Front. Microbiol..

[325] Li X., Zhang S., Guo G., Han J., Yu J. (2022). Gut microbiome in modulating immune checkpoint inhibitors. eBioMedicine.

[326] Petrosino J.F. (2018). The microbiome in precision medicine: The way forward. Genome Med..

[327] Haraoui L.-P., Blaser M.J. (2023). The Microbiome and Infectious Diseases. Clin. Infect. Dis..

[328] Hajjo R., Sabbah D.A., Al Bawab A.Q. (2022). Unlocking the Potential of the Human Microbiome for Identifying Disease Diagnostic Biomarkers. Diagnostics.

[329] Tribolet L., Kerr E., Cowled C., Bean A.G.D., Stewart C.R., Dearnley M., Farr R.J. (2020). MicroRNA Biomarkers for Infectious Diseases: From Basic Research to Biosensing. Front. Microbiol..

[330] Harrison X.A., Price S.J., Hopkins K., Leung W.T.M., Sergeant C., Garner T.W.J. (2019). Diversity-Stability Dynamics of the Amphibian Skin Microbiome and Susceptibility to a Lethal Viral Pathogen. Front. Microbiol..

[331] Yan J.-Y., Lin T.-H., Jong Y.-T., Hsueh J.-W., Wu S.-H., Lo H.-J., Chen Y.-C., Pan C.-H. (2024). Microbiota signatures associated with invasive Candida albicans infection in the gastrointestinal tract of immunodeficient mice. Front. Cell. Infect. Microbiol..

[332] Wallace R.K. (2020). The Microbiome in Health and Disease from the Perspective of Modern Medicine and Ayurveda. Medicina.

[333] Weersma R.K., Zhernakova A., Fu J. (2020). Interaction between drugs and the gut microbiome. Gut.

[334] Seong H., Choi B.K., Han Y.-H., Kim J.H., Gim J.-A., Lim S., Noh J.Y., Cheong H.J., Kim W.J., Song J.Y. (2023). Gut microbiota as a potential key to modulating humoral immunogenicity of new platform COVID-19 vaccines. Signal Transduct. Target. Ther..

[335] Jordan A., Carding S.R., Hall L.J. (2022). The early-life gut microbiome and vaccine efficacy. Lancet Microbe.

[336] Razzak M.I., Imran M., Xu G. (2020). Big data analytics for preventive medicine. Neural Comput. Appl..

[337] National Academies of Sciences, Engineering, and Medicine, Health and Medicine Division, Board on Global Health, Forum on Microbial Threats (2016). Opportunities and Challenges for Big Data and Analytics. Big Data and Analytics for Infectious Disease Research, Operations, and Policy: Proceedings of a Workshop.

[338] Yang X., Huang K., Yang D., Zhao W., Zhou X. (2024). Biomedical Big Data Technologies, Applications, and Challenges for Precision Medicine: A Review. Glob. Chall..

[339] Albahlal B.M. (2023). Emerging Technology-Driven Hybrid Models for Preventing and Monitoring Infectious Diseases: A Comprehensive Review and Conceptual Framework. Diagnostics.

[340] Begum R. (2016). A roadmap to translating the microbiome. Genome Med..

[341] Lamb C.A., Saifuddin A., Powell N., Rieder F. (2022). The Future of Precision Medicine to Predict Outcomes and Control Tissue Remodeling in Inflammatory Bowel Disease. Gastroenterology.

[342] Lukiw W.J. (2020). Human gastrointestinal (GI) tract microbiome-derived pro-inflammatory neurotoxins from Bacteroides fragilis: Effects of low fiber diets and environmental and lifestyle factors. Integr. Food Nutr. Metab..

[343] Murillo T., Schneider D., Heistermann M., Daniel R., Fichtel C. (2022). Assessing the drivers of gut microbiome composition in wild redfronted lemurs via longitudinal metacommunity analysis. Sci. Rep..

[344] Andersen L.O., Vedel Nielsen H., Stensvold C.R. (2013). Waiting for the human intestinal Eukaryotome. ISME J..

[345] Ram R.J., Verberkmoes N.C., Thelen M.P., Tyson G.W., Baker B.J., Blake R.C., Shah M., Hettich R.L., Banfield J.F. (2005). Community proteomics of a natural microbial biofilm. Science.

[346] Urich T., Lanzén A., Qi J., Huson D.H., Schleper C., Schuster S.C. (2008). Simultaneous assessment of soil microbial community structure and function through analysis of the meta-transcriptome. PLoS ONE.

[347] Benítez-Páez A., Sanz Y. (2017). Multi-locus and long amplicon sequencing approach to study microbial diversity at species level using the MinION™ portable nanopore sequencer. Gigascience.

[348] Ha C.W.Y., Devkota S. (2020). The new microbiology: Cultivating the future of microbiome-directed medicine. Am. J. Physiol. Gastrointest. Liver Physiol..

[349] Harris V.C., Haak B.W., van Boele Hensbroek M., Wiersinga W.J. (2017). The Intestinal Microbiome in Infectious Diseases: The Clinical Relevance of a Rapidly Emerging Field. Open Forum Infect. Dis..

[350] Zhang Z.J., Lehmann C.J., Cole C.G., Pamer E.G. (2022). Translating Microbiome Research from and To the Clinic. Annu. Rev. Microbiol..

[351] Berg G., Rybakova D., Fischer D., Cernava T., Vergès M.-C.C., Charles T., Chen X., Cocolin L., Eversole K., Corral G.H. (2020). Microbiome definition re-visited: Old concepts and new challenges. Microbiome.

[352] Szóstak N., Szymanek A., Havránek J., Tomela K., Rakoczy M., Samelak-Czajka A., Schmidt M., Figlerowicz M., Majta J., Milanowska-Zabel K. (2022). The standardisation of the approach to metagenomic human gut analysis: From sample collection to microbiome profiling. Sci. Rep..

[353] Papoutsoglou G., Tarazona S., Lopes M.B., Klammsteiner T., Ibrahimi E., Eckenberger J., Novielli P., Tonda A., Simeon A., Shigdel R. (2023). Machine learning approaches in microbiome research: Challenges and best practices. Front. Microbiol..

[354] Matijašić M., Meštrović T., Paljetak H.Č., Perić M., Barešić A., Verbanac D. (2020). Gut Microbiota beyond Bacteria-Mycobiome, Virome, Archaeome, and Eukaryotic Parasites in IBD. Int. J. Mol. Sci..

[355] Ghosh T.S., Shanahan F., O’Toole P.W. (2022). The gut microbiome as a modulator of healthy ageing. Nat. Rev. Gastroenterol. Hepatol..

[356] Odamaki T., Kato K., Sugahara H., Hashikura N., Takahashi S., Xiao J.-Z., Abe F., Osawa R. (2016). Age-related changes in gut microbiota composition from newborn to centenarian: A cross-sectional study. BMC Microbiol..

[357] Rizzetto L., Fava F., Tuohy K.M., Selmi C. (2018). Connecting the immune system, systemic chronic inflammation and the gut microbiome: The role of sex. J. Autoimmun..

[358] Jackson D., Maltz M.R., Freund H.L., Borneman J., Aronson E. (2021). Environment and Diet Influence the Bacterial Microbiome of *Ambigolimax valentianus*, an Invasive Slug in California. Insects.

[359] Kwan S.-Y., Sabotta C.M., Joon A., Wei P., Petty L.E., Below J.E., Wu X., Zhang J., Jenq R.R., Hawk E.T. (2022). Gut Microbiome Alterations Associated with Diabetes in Mexican Americans in South Texas. mSystems.

[360] Harris E.V., de Roode J.C., Gerardo N.M. (2019). Diet-microbiome-disease: Investigating diet’s influence on infectious disease resistance through alteration of the gut microbiome. PLoS Pathog..

[361] Aguiar-Pulido V., Huang W., Suarez-Ulloa V., Cickovski T., Mathee K., Narasimhan G. (2016). Metagenomics, Metatranscriptomics, and Metabolomics Approaches for Microbiome Analysis. Evol. Bioinform..

[362] Liu Z., Ma A., Mathé E., Merling M., Ma Q., Liu B. (2021). Network analyses in microbiome based on high-throughput multi-omics data. Brief. Bioinform..

[363] Gilbert J.A., Blaser M.J., Caporaso J.G., Jansson J.K., Lynch S.V., Knight R. (2018). Current understanding of the human microbiome. Nat. Med..

[364] Shreiner A.B., Kao J.Y., Young V.B. (2015). The gut microbiome in health and in disease. Curr. Opin. Gastroenterol..

[365] Kachroo N., Lange D., Penniston K.L., Stern J., Tasian G., Bajic P., Wolfe A.J., Suryavanshi M., Ticinesi A., Meschi T. (2021). Standardization of microbiome studies for urolithiasis: An international consensus agreement. Nat. Rev. Urol..

[366] Patangia D.V., Anthony Ryan C., Dempsey E., Paul Ross R., Stanton C. (2022). Impact of antibiotics on the human microbiome and consequences for host health. MicrobiologyOpen.

[367] Hrncir T. (2022). Gut Microbiota Dysbiosis: Triggers, Consequences, Diagnostic and Therapeutic Options. Microorganisms.

[368] Dahiya D., Nigam P.S. (2023). Antibiotic-Therapy-Induced Gut Dysbiosis Affecting Gut Microbiota-Brain Axis and Cognition: Restoration by Intake of Probiotics and Synbiotics. Int. J. Mol. Sci..

[369] Zhang Y., Zhang H., Xu T., Zeng L., Liu F., Huang X., Liu Q. (2022). Interactions among microorganisms open up a new world for anti-infectious therapy. FEBS J..

[370] Smith H. (1982). The role of microbial interactions in infectious disease. Philos. Trans. R. Soc. Lond. B Biol. Sci..

[371] O’Daniel M., Rosenstein A.H., O’Daniel M., Rosenstein A.H. (2008). Professional Communication and Team Collaboration. Patient Safety and Quality: An Evidence-Based Handbook for Nurses.

[372] Slashinski M.J., Whitney S.N., Achenbaum L.S., Keitel W.A., McCurdy S.A., McGuire A.L. (2013). Investigators’ perspectives on translating human microbiome research into clinical practice. Public Health Genom..

[373] Grieneisen L., Blekhman R., Archie E. (2023). How longitudinal data can contribute to our understanding of host genetic effects on the gut microbiome. Gut Microbes.

[374] Bernard G., Pathmanathan J.S., Lannes R., Lopez P., Bapteste E. (2018). Microbial Dark Matter Investigations: How Microbial Studies Transform Biological Knowledge and Empirically Sketch a Logic of Scientific Discovery. Genome Biol. Evol..

[375] Zamkovaya T., Foster J.S., de Crécy-Lagard V., Conesa A. (2021). A network approach to elucidate and prioritize microbial dark matter in microbial communities. ISME J..

[376] Schultz J., Modolon F., Peixoto R.S., Rosado A.S. (2023). Shedding light on the composition of extreme microbial dark matter: Alternative approaches for culturing extremophiles. Front. Microbiol..

[377] Abdul-Aziz M.A., Cooper A., Weyrich L.S. (2016). Exploring Relationships between Host Genome and Microbiome: New Insights from Genome-Wide Association Studies. Front. Microbiol..

[378] Sarkar A., Yoo J.Y., Valeria Ozorio Dutra S., Morgan K.H., Groer M. (2021). The Association between Early-Life Gut Microbiota and Long-Term Health and Diseases. J. Clin. Med..

[379] Stiemsma L.T., Michels K.B. (2018). The Role of the Microbiome in the Developmental Origins of Health and Disease. Pediatrics.

[380] Lynch S.V., Pedersen O. (2016). The Human Intestinal Microbiome in Health and Disease. N. Engl. J. Med..

[381] Fujimura K.E., Demoor T., Rauch M., Faruqi A.A., Jang S., Johnson C.C., Boushey H.A., Zoratti E., Ownby D., Lukacs N.W. (2014). House dust exposure mediates gut microbiome Lactobacillus enrichment and airway immune defense against allergens and virus infection. Proc. Natl. Acad. Sci. USA.

[382] Ellabaan M.M.H., Munck C., Porse A., Imamovic L., Sommer M.O.A. (2021). Forecasting the dissemination of antibiotic resistance genes across bacterial genomes. Nat. Commun..

[383] Garmaeva S., Sinha T., Kurilshikov A., Fu J., Wijmenga C., Zhernakova A. (2019). Studying the gut virome in the metagenomic era: Challenges and perspectives. BMC Biol..

[384] Singh R.K., Chang H.-W., Yan D., Lee K.M., Ucmak D., Wong K., Abrouk M., Farahnik B., Nakamura M., Zhu T.H. (2017). Influence of diet on the gut microbiome and implications for human health. J. Transl. Med..

[385] Zhang P. (2022). Influence of Foods and Nutrition on the Gut Microbiome and Implications for Intestinal Health. Int. J. Mol. Sci..

[386] Durack J., Lynch S.V. (2019). The gut microbiome: Relationships with disease and opportunities for therapy. J. Exp. Med..

[387] Fröhlich H., Balling R., Beerenwinkel N., Kohlbacher O., Kumar S., Lengauer T., Maathuis M.H., Moreau Y., Murphy S.A., Przytycka T.M. (2018). From hype to reality: Data science enabling personalized medicine. BMC Med..

[388] Conlon M.A., Bird A.R. (2014). The impact of diet and lifestyle on gut microbiota and human health. Nutrients.

[389] Ren Y., Wu J., Wang Y., Zhang L., Ren J., Zhang Z., Chen B., Zhang K., Zhu B., Liu W. (2023). Lifestyle patterns influence the composition of the gut microbiome in a healthy Chinese population. Sci. Rep..

[390] Vijay A., Valdes A.M. (2022). Role of the gut microbiome in chronic diseases: A narrative review. Eur. J. Clin. Nutr..

[391] Bengtsson-Palme J. (2020). Microbial model communities: To understand complexity, harness the power of simplicity. Comput. Struct. Biotechnol. J..

[392] Song H.-S., Cannon W., Beliaev A., Konopka A. (2014). Mathematical Modeling of Microbial Community Dynamics: A Methodological Review. Processes.

[393] Shi A., Fan F., Broach J.R. (2022). Microbial adaptive evolution. J. Ind. Microbiol. Biotechnol..

[394] Henry L.P., Bruijning M., Forsberg S.K.G., Ayroles J.F. (2021). The microbiome extends host evolutionary potential. Nat. Commun..

[395] Tan Y.-S., Zhang R.-K., Liu Z.-H., Li B.-Z., Yuan Y.-J. (2022). Microbial Adaptation to Enhance Stress Tolerance. Front. Microbiol..

[396] Relman D.A. (2011). Microbial genomics and infectious diseases. N. Engl. J. Med..

[397] Lange L., Berg G., Cernava T., Champomier-Vergès M.-C., Charles T., Cocolin L., Cotter P., D’Hondt K., Kostic T., Maguin E. (2022). Microbiome ethics, guiding principles for microbiome research, use and knowledge management. Environ. Microbiome.

[398] Todd O.A., Peters B.M. (2019). Candida albicans and Staphylococcus aureus Pathogenicity and Polymicrobial Interactions: Lessons beyond Koch’s Postulates. J. Fungi.

[399] Manna S., Weinberger D.M., Satzke C. (2023). Editorial: Thematic issue on bacterial-viral co-infections. FEMS Microbes.

[400] Li D., Gao C., Zhang F., Yang R., Lan C., Ma Y., Wang J. (2020). Seven facts and five initiatives for gut microbiome research. Protein Cell.

[401] Sharma V.K., Singh T.G., Garg N., Dhiman S., Gupta S., Rahman M.H., Najda A., Walasek-Janusz M., Kamel M., Albadrani G.M. (2021). Dysbiosis and Alzheimer’s Disease: A Role for Chronic Stress?. Biomolecules.

[402] Spivak I., Fluhr L., Elinav E. (2022). Local and systemic effects of microbiome-derived metabolites. EMBO Rep..

[403] Graham D.B., Xavier R.J. (2023). Conditioning of the immune system by the microbiome. Trends Immunol..

[404] Tomaiuolo R., Veneruso I., Cariati F., D’Argenio V. (2020). Microbiota and Human Reproduction: The Case of Female Infertility. High-Throughput.

[405] Grobeisen-Duque O., Mora-Vargas C.D., Aguilera-Arreola M.G., Helguera-Repetto A.C. (2023). Cycle Biodynamics of Women’s Microbiome in the Urinary and Reproductive Systems. J. Clin. Med..

[406] Sklyar T.V., Voronkova O.S., Krysenko O.V., Papiashvili M.H., Shevchenko T.M., Vinnikov A.I. (2016). Association of microorganisms of reproductive tract of women with vaginal microbiome disorders. Microbiol. Med..

[407] Chotirmall S.H., Gellatly S.L., Budden K.F., Mac Aogain M., Shukla S.D., Wood D.L.A., Hugenholtz P., Pethe K., Hansbro P.M. (2017). Microbiomes in respiratory health and disease: An Asia-Pacific perspective. Respirology.

[408] Belkaid Y., Segre J.A. (2014). Dialogue between skin microbiota and immunity. Science.

[409] Celoria V., Rosset F., Pala V., Dapavo P., Ribero S., Quaglino P., Mastorino L. (2023). The Skin Microbiome and Its Role in Psoriasis: A Review. Psoriasis.

[410] Geng J., Ni Q., Sun W., Li L., Feng X. (2022). The links between gut microbiota and obesity and obesity related diseases. Biomed. Pharmacother..

[411] Kozak M., Pawlik A. (2023). The Role of the Oral Microbiome in the Development of Diseases. Int. J. Mol. Sci..

[412] Wu H.-J., Wu E. (2012). The role of gut microbiota in immune homeostasis and autoimmunity. Gut Microbes.

[413] Grundmann O. (2020). Gastrointestinal Inflammation and the Gut Microbiome: An Evolving Conceptual Framework with Implications for Diagnosis and Therapy in Inflammatory Bowel Disorders. EMJ Microbiol Infect Dis..

[414] Aboushaala K., Wong A.Y.L., Barajas J.N., Lim P., Al-Harthi L., Chee A., Forsyth C.B., Oh C., Toro S.J., Williams F.M.K. (2023). The Human Microbiome and Its Role in Musculoskeletal Disorders. Genes..

[415] Xu Z., Xie Z., Sun J., Huang S., Chen Y., Li C., Sun X., Xia B., Tian L., Guo C. (2020). Gut Microbiome Reveals Specific Dysbiosis in Primary Osteoporosis. Front. Cell. Infect. Microbiol..

[416] Liu C., Cheung W.-H., Li J., Chow S.K.-H., Yu J., Wong S.H., Ip M., Sung J.J.Y., Wong R.M.Y. (2021). Understanding the gut microbiota and sarcopenia: A systematic review. J. Cachexia Sarcopenia Muscle.

[417] Zhang J., Lu Y., Wang Y., Ren X., Han J. (2018). The impact of the intestinal microbiome on bone health. Intractable Rare Dis. Res..

[418] Chew W., Lim Y.P., Lim W.S., Chambers E.S., Frost G., Wong S.H., Ali Y. (2022). Gut-muscle crosstalk. A perspective on influence of microbes on muscle function. Front. Med..

